# Fruit and Vegetable By-Products as Postharvest Tissue Fractions: A Raw-Material Framework for Plant-Based Food Development

**DOI:** 10.3390/plants15162423

**Published:** 2026-08-08

**Authors:** Hyo Jun Won, Ae-jin Choi

**Affiliations:** Food Tech Resources Research Division, National Institute of Crop and Food Science (NICS), Rural Development Administration (RDA), Wanju-gun 55365, Republic of Korea

**Keywords:** fruit and vegetable by-products, postharvest tissue fractions, tissue segregation, stabilization, safety screening, raw-material specifications, Brassicaceae, analytical fingerprinting, plant-based food ingredients, zero-waste route assignment

## Abstract

Fruit and vegetable by-products can be interpreted more usefully as postharvest crop-derived tissue fractions than as generic waste streams or sources of recoverable compounds. This integrative review synthesizes evidence on plant tissues, postharvest quality, stabilization, safety, and analytical characterization to develop a tissue-to-raw-material framework for plant-based food development. The framework begins with crop source, cultivar or maturity, organ/tissue identity, and postharvest history, and then links these variables to stabilization, safety screening, analytical evidence, intended-use specifications, and route assignment. Brassicaceae crops—including napa cabbage, radish, cabbage, broccoli, and cauliflower—serve as representative cases because their leafy, root, stem, core, stalk, and trimming fractions differ in water status, tissue fragility, sulfur-related traits, sensory constraints, and stabilization needs. Application suitability is therefore assessed from tissue identity, postharvest condition, stabilization history, safety status, and intended-use specifications rather than compound richness alone. Within this framework, zero-waste development means assigning a documented tissue fraction to a supported food, fermentation, coating/film, or selected secondary-material route, while using lower-risk assignment, downgrading, or exclusion where traceability, safety, stability, or specification evidence remains insufficient.

## 1. Introduction

Fruit and vegetable value chains generate diverse side-streams during harvesting, trimming, sorting, fresh-cut handling, storage, and processing. In crop-quality terms, these materials are not merely end-of-chain losses; many remain identifiable as crop-derived organs or tissue fractions whose quality history can still be traced, documented, and managed [1,2,3]. Their value therefore depends not only on recoverable compounds or final formulations, but also on botanical source, organ identity, tissue condition, and postharvest physiology.

Peels, pomace, seeds, press cakes, outer leaves, stems, roots, trimmings, and sorting residues differ in anatomy, water status, cell-wall organization, phytochemical localization, respiration potential, microbial exposure, and damage history. They may contain dietary fiber, pectin, cellulose, hemicellulose, minerals, pigments, phenolics, carotenoids, glucosinolates, proteins, and other application-relevant constituents [4,5,6]. However, compound richness alone is insufficient to demonstrate raw-material suitability. The same constituent profile can lead to different stability, safety, sensory, and processing outcomes depending on tissue structure, moisture status, damage history, stabilization conditions, and intended route [2,4,7].

Plant tissue architecture provides the biological basis for this interpretation. Epidermal and cuticular barriers, parenchyma, vascular tissues, storage and protective tissues, cell-wall networks, water-rich cellular compartments, and localized phytochemical systems influence water loss, softening, browning, lignification, pigment degradation, sulfur-related odor formation, microbial susceptibility, drying behavior, milling behavior, hydration properties, and sensory constraints [8,9,10,11]. Therefore, a tissue-centered review should ask not only which useful compounds are present but also under what postharvest, stabilization, safety, and specification conditions a crop-derived tissue fraction can be preserved, standardized, and assigned to an appropriate route.

Previous by-product reviews have clarified recovery technologies, bioactive fractions, circular valorization routes, and downstream food or material applications [5,12,13]. Building on this literature, the present review adds an upstream plant-tissue and postharvest-quality layer rather than replacing extraction-, compound-, circular-economy-, fermentation-, food-ingredient-, or material-route reviews. The proposed tissue-to-raw-material framework links crop source, organ/tissue fraction, quality history, stabilization, analytical evidence, safety status, raw-material specifications, and specification-based route assignment. Here, zero-waste development denotes evidence-based route assignment based on traceability, safety, stability, and specifications for the intended use; it does not imply unconditional reuse.

This integrative review focuses on fruit and vegetable by-products generated across production, postharvest handling, sorting, fresh-cut processing, and industrial processing. Brassicaceae crops, including napa cabbage, radish, cabbage, broccoli, and cauliflower, are used as representative case examples rather than as an exclusive taxonomic focus, because leafy, root, stem, core, stalk, and trimming fractions differ in water status, tissue fragility, sulfur-related traits, odor constraints, and stabilization needs [6,14,15]. Accordingly, this review examines how tissue-level and postharvest traits define raw-material identity, how stabilization, safety screening, and analytical evidence convert unstable fractions into specification-based candidates, and how those candidates can be assigned conservatively to plant-based food or selected secondary-material routes.

## 2. Review Approach and Evidence-Mapping Strategy

Because the evidence spans plant anatomy, postharvest physiology, stabilization, chemical characterization, safety, and application studies that are heterogeneous in design and outcome, we used an integrative review supported by a structured evidence-mapping strategy [16]. Here, evidence mapping refers to the organization of the heterogeneous literature across predefined conceptual domains rather than to a formal scoping-review map or quantitative synthesis. Search domains, eligibility criteria, and synthesis logic were explicitly defined to make the integration transparent without characterizing the review as a systematic or scoping review [17,18,19].

### 2.1. Information Sources and Search Strategy

The evidence synthesis was developed iteratively during manuscript preparation. To establish the final reproducible source record and exact evidence-flow counts, the Scopus and Web of Science Core Collection searches, together with Google Scholar queries GS_Q1–GS_Q4, were run on 28 July 2026. The PubMed search and Google Scholar query GS_Q5 were run on 29 July 2026; the Web of Science search was rerun on the same date solely to verify the exact syntax and displayed result count, while the original 28 July export was retained. Google Scholar searches were conducted in a logged-out Microsoft Edge InPrivate session, using relevance ordering and no date or language restriction. The prespecified retrieval depth was the first 50 accessible relevance-ranked results per query; 50, 50, 50, 43, and 34 results were inspected and captured, respectively, yielding 227 raw Google Scholar records for the merged source pool. This retrieval-stage inspection determined which Google Scholar records entered the pool and was distinct from the subsequent title/abstract eligibility screening of deduplicated records. Patents and citation-only records were excluded. Estimated Google Scholar hit counts were retained only as internal audit metadata and were not used in the evidence-flow accounting. Backward and forward citation searching were not performed. The final source search was completed on 29 July 2026.

After completion of the final source search, records from all sources were consolidated in the master evidence-flow workbook. Duplicate relations were resolved, and the resulting unique-record pool was reconciled with the retained title/abstract and full-text assessment logs. H.J.W. finalized the record-management and eligibility records, A.-j.C. verified the recorded screening and full-text outcomes, and the consolidated evidence-flow record was finalized and author-verified on 30 July 2026.

The source-specific strategies combined by-product and tissue-fraction terms with crop-source and postharvest stabilization, specification, characterization, and application concepts. Complete platform-specific search strings, field codes, limits, search dates, retrieval counts, and Google Scholar handling rules are reported in Supplementary Table S3.

Before deduplication, 1681 records were identified: 699 from Scopus, 192 from PubMed, 563 from Web of Science Core Collection, and 227 from Google Scholar. After 679 duplicate records were removed, 1002 unique records underwent title/abstract screening. Thirty-three records were excluded at this stage, and 969 reports were sought and retrieved for full-text assessment; no report was unavailable. Ninety-seven reports were excluded after full-text assessment, leaving 872 eligible reports in the evidence bank.

The 134-item manuscript reference list was not a simple subset of the 872-report evidence bank and was not generated by an additional eligibility screen. The evidence bank retained all reports meeting at least one predefined evidence domain. The manuscript reference list combined eligible evidence-bank reports cited directly for specific statements, tables, figures, or synthesis domains with contextual, methodological, regulatory, and reporting-policy sources used where needed. The criteria for individual citation are described in Section 2.3 and Supplementary Table S3.

Each full-text exclusion was assigned one mutually exclusive primary reason: outside material scope (FT1, n = 0), insufficient tissue identity (FT2, n = 0), no tissue-to-raw-material link (FT3, n = 0), pharmacological or clinical focus without material-level relevance (FT4, n = 0), energy-recovery or disposal focus without raw-material relevance (FT5, n = 0), or insufficient usable information for the predefined synthesis domains (FT6, n = 97). Material-scope, tissue-identity, and clearly non-material-focused records were resolved mainly during title/abstract screening; consequently, all reports excluded after full-text assessment were assigned to FT6. The complete selection flow is shown in Supplementary Figure S1.

### 2.2. Inclusion and Exclusion Criteria

The inclusion and exclusion criteria were applied during both title/abstract screening and full-text assessment to determine whether each record could contribute to the tissue-to-raw-material synthesis. Records were retained if they provided sufficient information to link fruit- or vegetable-derived materials with at least one predefined evidence domain, such as tissue identity, postharvest quality, stabilization, safety/specification evidence, or application context.

Studies and reviews were eligible if they addressed fruit- or vegetable-derived plant materials and contributed to at least one predefined evidence domain. These domains included organ or tissue identity, postharvest quality, stabilization, drying or milling behavior, compositional characterization, safety, functional properties, raw-material standardization, downstream plant-based food development, or selected secondary-material routes. Particular attention was given to reports that specified crop species, cultivar or genotype, organ fraction, tissue type, maturity, processing point, postharvest handling, or storage conditions because these variables directly affect the interpretation of by-products as plant tissues.

Studies were excluded if they focused solely on isolated pharmacological activity without adequate information on plant source or tissue fraction, or if they used residues only as generic substrates for energy recovery or disposal. Studies of animal-derived by-products, cereal-milling residues, municipal waste, clinical health claims, or final food formulations were also excluded unless they provided a clear link to tissue-derived raw-material properties. Official reports and policy documents were used only for terminology, food loss/waste context, safety framing, or circular-economy background, not as substitutes for peer-reviewed plant, postharvest, or material-quality evidence.

During full-text assessment, each excluded report was assigned one mutually exclusive primary reason, while reports that could not be retrieved were recorded separately. The six exclusion categories and their reason-specific counts are shown in Supplementary Figure S1 and described in Supplementary Table S3.

H.J.W. completed and confirmed deduplication, title/abstract screening, full-text assessment, exclusion-reason assignment, and reference checking. A.-j.C. verified the recorded screening and full-text eligibility outcomes, and disagreements were resolved through discussion. The final decision, primary reason (where applicable), assessor, verifier, and decision date were retained in the audit log. Search-string development, metadata handling, organization of potential duplicate records, and preparation of the search and screening logs were performed by the authors. Generative AI tools were not used to select evidence, generate bibliographic references, resolve duplicates, conduct title/abstract or full-text screening, determine eligibility, assign exclusion reasons, classify evidence, assess study quality, interpret findings, verify references, or make methodological or scientific decisions. All decisions concerning duplicate resolution, eligibility, exclusion-reason assignment, evidence classification, interpretation, and reference verification were made and confirmed by the authors.

All 134 references cited in the manuscript were manually checked using the publisher’s version of record, the corresponding DOI landing page, PubMed where applicable, or the relevant official institutional source. The audit covered the complete author list, article title, journal title, publication year, volume and issue where applicable, page range or article number, and DOI or official URL. Quantitative values, analytical bases, processing conditions, and claim boundaries in Supplementary Table S1 were independently cross-checked against the cited primary reports.

### 2.3. Synthesis Logic

Evidence was synthesized across seven axes: crop source, tissue fraction, postharvest state, stabilization, compositional or structural profile, safety and specification status, and application context. Between-study disagreement was interpreted first as possible heterogeneity in crop, cultivar, maturity, tissue segregation, postharvest interval, stabilization method, analytical platform, or target route rather than as simple inconsistency [2,4,9,14].

Greater interpretive weight was assigned to relationships supported across multiple crop or tissue contexts or by convergent biological and processing evidence. Findings derived mainly from screening assays, single materials, poorly defined streams, or incomplete postharvest and safety records were interpreted as context-dependent. Each finding was then classified as screening-level evidence, specification-supporting evidence, or route-level validation. Screening-level evidence denotes exploratory or incompletely defined observations; specification-supporting evidence requires transparent material identity, process conditions, analytical methods, units, and claim boundaries; and route-level validation requires performance in the intended application together with adequate safety, stability, and process-suitability evidence.

Individual citation was determined after evidence-bank eligibility and did not represent a second inclusion decision. Evidence-bank reports were cited when they provided direct, non-redundant support for a specific statement, table, figure, or synthesis domain. Where several eligible reports supported substantially the same point, priority was given to sources with clearly reported crop and tissue identity, postharvest or processing conditions, analytical methods and units, explicit claim boundaries, and the closest relevance to the statement being supported. Additional sources were cited when they supplied non-redundant crop, tissue, stabilization, safety, specification, or application context. Primary studies were generally prioritized for quantitative and process-specific claims, broader reviews for cross-cutting synthesis, and official sources for methodological, regulatory, or policy statements. Eligible reports not cited individually remained in the evidence bank and were not treated as additional exclusions.

### 2.4. Distinction from Related Review Streams and Scope Control

**Figure 1:**
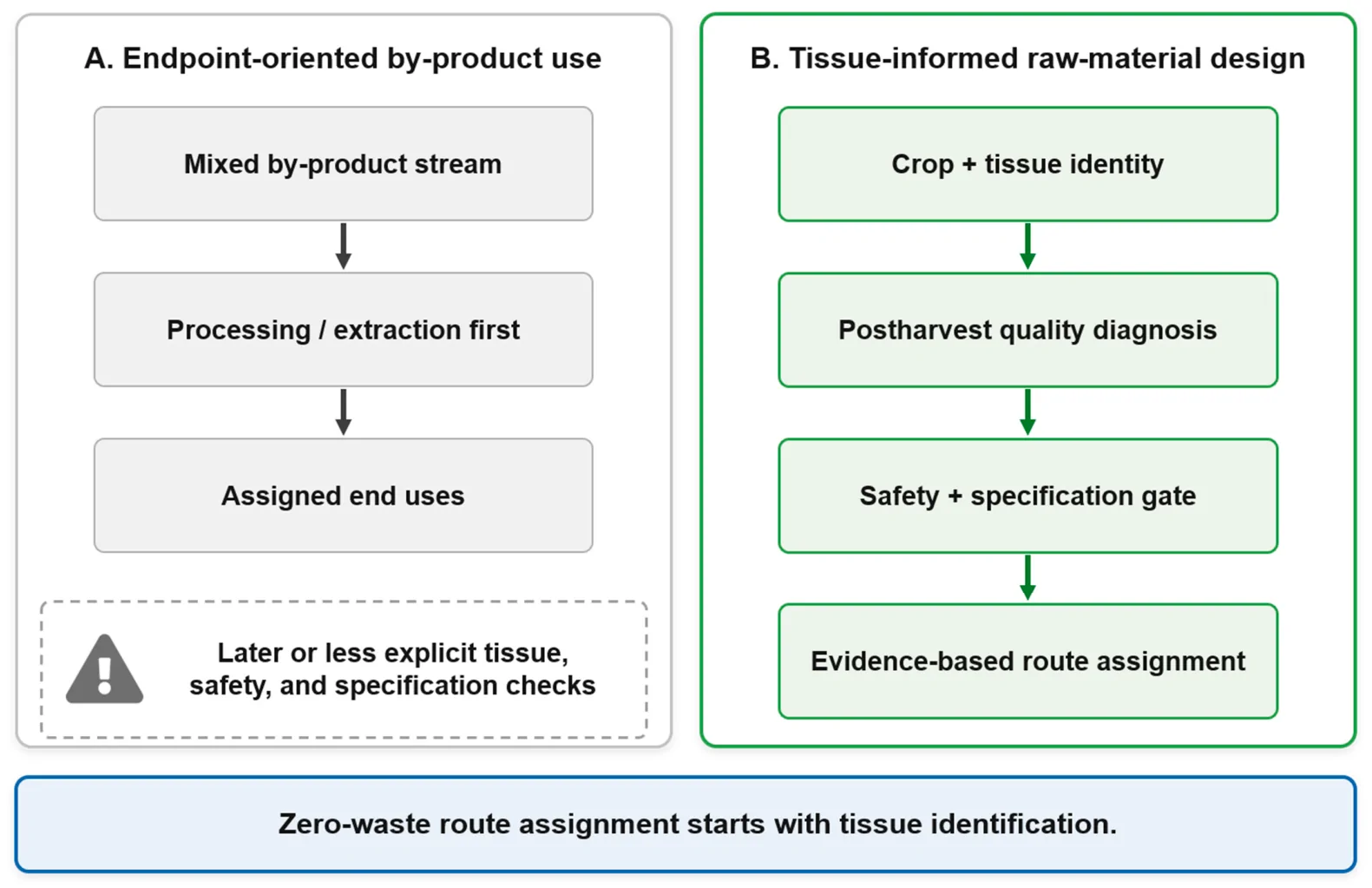
Conceptual shift from endpoint-oriented by-product use to tissue-informed raw-material design. (**A**) Endpoint-oriented approaches often begin with mixed by-product streams and move toward processing, extraction, or assigned end uses; tissue identity, postharvest history, and safety/specification checks may be considered later or less explicitly. (**B**) The tissue-informed approach starts with crop and tissue identity, diagnoses postharvest quality, applies safety and specification gates, and then assigns routes according to evidence.

Extraction, bioactivity, fermentation, coating/film, and material studies were included only when they informed tissue identity, postharvest quality, stabilization, safety, specifications, or route assignment. Accordingly, the unit of synthesis was the crop–organ–tissue fraction and its quality trajectory rather than the extraction technology, isolated compound class, or final product category. The framework adds an upstream tissue-identity and postharvest-quality layer to existing extraction-, compound-, circular-economy-, fermentation-, food-ingredient-, and material-route reviews. The positioning of the present tissue-informed review relative to representative review streams is summarized in Table 1. The conceptual shift from endpoint-oriented by-product use to tissue-informed raw-material design is illustrated in Figure 1.

The present review complements these streams by adding an upstream tissue identity, postharvest quality, stabilization, safety, and specification layer.

## 3. Conceptual Reframing: Postharvest Plant Tissues as Crop-Quality Resources

Fruit and vegetable by-products are often introduced as waste streams, residues, or low-value biomass, but these terms can obscure their remaining organ-specific structure, metabolism, composition, and deterioration behavior. A tissue-centered view is useful because by-products generated after harvest, trimming, sorting, fresh-cut handling, or industrial processing often remain anatomically recognizable and physiologically labile. Accordingly, these materials are evaluated as postharvest plant tissues according to their fraction identity, retained quality state, and the evidence supporting a safe and reproducible application pathway.

### 3.1. Defining Waste, Residue, By-Product, Side-Stream, and Raw Material

In this review, “waste” denotes plant material that is discarded or must be discarded, whereas “residue” denotes material remaining after production or processing. “By-product” denotes a production-derived fraction for which further lawful use may be considered, and “side-stream” describes a parallel material flow without implying legal, safety, or quality status [3,37,38,39]. A “raw-material candidate” is a traceable tissue fraction that has undergone defined stabilization and preliminary characterization but still lacks one or more intended-use specifications, safety criteria, storage data, or application-validation requirements. “Raw material” is reserved for a stabilized and characterized input that meets the identity, safety, stability, and performance specifications defined for its intended use. An “application pathway” is a broad use category, whereas “route assignment” is the evidence-based decision to select a supported pathway or a fallback outcome. “Routing” refers only to the implementation of an assigned route. These operational definitions do not determine legal status across jurisdictions.

### 3.2. Organ Identity and Tissue-Level Heterogeneity in Fruit and Vegetable By-Products

Organ identity is the first practical discriminator among by-product fractions. Peels, pomace, seeds, outer leaves, stems, stalks, roots, cores, and trimmings differ in tissue architecture, water status, mechanical strength, microbial exposure, phytochemical localization, and sensory constraints [4,8,9,14]. Accordingly, outer leaves, radish roots, cabbage cores, broccoli stalks, and other recognizable fractions are interpreted as distinct materials rather than pooled within a generic vegetable-waste category.

### 3.3. Tissue Architecture, Processability, and Structure–Process Compatibility

Structure then constrains processing. Cuticular barriers, parenchyma, vascular bundles, lignification, cell-wall organization, and porosity affect moisture migration, shrinkage, rehydration, milling resistance, hydration, and texture [7,8,9,40]. Mechanical injury further changes these properties by disrupting compartmentation and accelerating water loss, browning, microbial attachment, softening, and odor development [9,41,42,43]. Thus, the meaning of a compositional marker depends on the tissue matrix in which it occurs.

### 3.4. Water Status, Respiration, Enzymatic Activity, and Spoilage Susceptibility

Water status and tissue injury define stabilization urgency. High-moisture or wounded fractions are prioritized for rapid time–temperature control, sanitation, or stabilization because respiration, enzymatic activity, sensory deterioration, and microbial growth can continue after separation [9,43]. Dense, fibrous tissues may deteriorate less visibly but require explicit assessment of size-reduction and drying feasibility. Route assignment follows only after the dominant instability has been identified.

### 3.5. Cultivar, Maturity, Growing Conditions, and Harvest Timing

Cultivar, maturity, production context, harvest timing, and postharvest interval function here as interpretation covariates rather than independent route criteria [10,11,44,45,46]. They should be reported when they plausibly explain tissue structure, marker composition, deterioration, or storage behavior. When they are missing, processing effects should be interpreted cautiously because source-related variation cannot be separated reliably from treatment effects.

## 4. Crop- and Tissue-Specific By-Product Classes and Their Raw-Material Potential

Fruit- and vegetable-derived fractions differ in organ identity, tissue architecture, water status, exposure history, and stabilization needs. Organizing the evidence by source and tissue fraction therefore prevents biologically distinct materials from being interpreted as equivalent and supports conservative assessment of stabilization priority, safety, specification evidence, and intended application [2,4,6,14].

### 4.1. Fruit-Derived By-Products

For synthesis, fruit-derived fractions are grouped as peel-rich, pomace/pulp-rich, and seed/press-cake materials. Peel-rich fractions are dominated by protective tissues and surface exposure; pomace and pulp residues are wet, mixed matrices whose behavior depends on the peel–pulp–seed ratio; seeds and press cakes are dense storage tissues requiring attention to oxidation, allergens, antinutritional factors, and milling. Route assignment for these groups requires evidence on stabilization, safety, and material behavior in addition to composition [2,4,25,26,47].

### 4.2. Vegetable-Derived By-Products

Vegetable-derived fractions are grouped as leafy tissues, stems/stalks/cores, and roots/trimmings. Leafy fractions are generally moisture-sensitive and exposed; structural fractions are dominated by fiber, texture, and particle behavior; root fractions combine storage-tissue properties with soil-contact and contaminant considerations [6,14,15,48]. This grouping defines screening priorities but does not imply uniform composition or route suitability within any class.

### 4.3. Brassicaceae as a Representative Case Study

*Brassicaceae* serve as a representative case study because their shared botanical background encompasses contrasting leafy, root, stem, core, stalk, inflorescence, and trimming fractions [6,14,15]. These tissues differ in moisture, fiber architecture, pigments, glucosinolate–myrosinase behavior, sulfur-related sensory traits, and stabilization urgency. The family therefore provides a compact test of whether tissue identity improves interpretation across related crops; it is not used to define the taxonomic limits of the framework.

Within this study, napa cabbage outer leaves were interpreted as rapidly deteriorating leafy tissues, radish root trimmings as soil-contact storage tissues, and broccoli stalks as fibrous structural tissues. Their potential value was judged from these tissue properties rather than from a shared Brassicaceae label or glucosinolate content alone.

### 4.4. Why Tissue Segregation Matters

Tissue segregation is a prerequisite for reproducible stabilization and specification. Pooling peels, leaves, stems, roots, and pomace increases variation in moisture, particle behavior, odor, fiber composition, microbial quality, and drying response; a mixed stream therefore supports fewer specific claims than a traceable tissue fraction [4,14,27].

**Figure 2:**
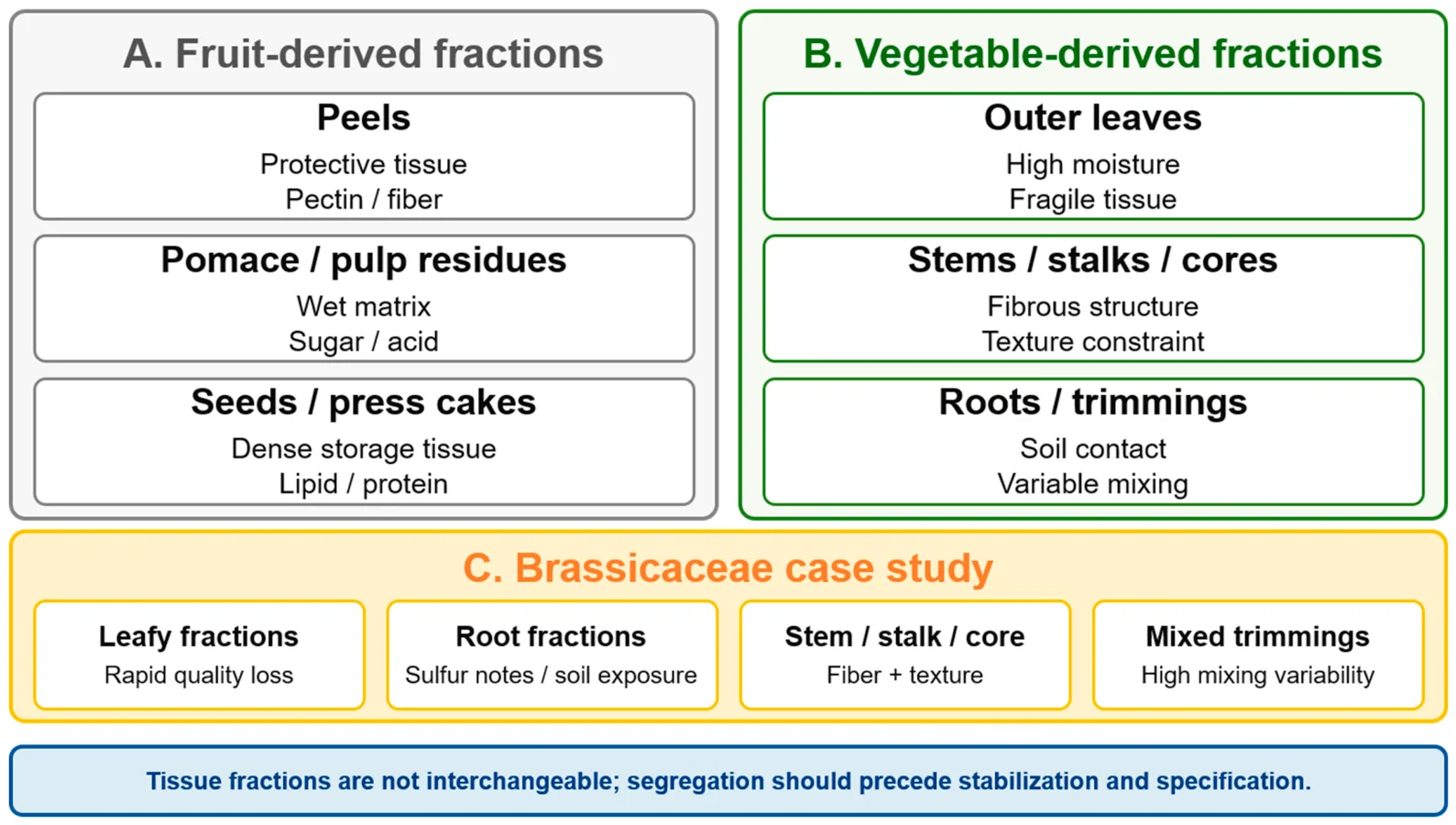
Source- and tissue-specific heterogeneity of fruit and vegetable by-product fractions. (**A**) Fruit-derived fractions are condensed into peel-rich, pomace/pulp-rich, and seed/press-cake fractions because these groups differ in protective tissue structure, wet matrix behavior, and dense storage-tissue composition. (**B**) Vegetable-derived fractions are condensed into leafy, stem/stalk/core, and root/trimming fractions, which differ in moisture load, fibrous structure, soil-contact exposure, sensory constraints, and mixing status. (**C**) Brassicaceae residues are used as a representative case study to show that leafy, root, stem/stalk/core, and mixed trimming fractions should be interpreted as distinct tissue fractions before stabilization and specification.

Broccoli stalks and cabbage outer leaves illustrate this decision. Stalks are prioritized for size reduction, fiber, texture, and particle behavior evaluation; outer leaves are prioritized for rapid stabilization, sanitation, microbial and contaminant screening, and sensory assessment. We therefore do not treat the two fractions as interchangeable Brassicaceae residues, even when both contain potentially useful fiber or phytochemicals. The source- and tissue-specific heterogeneity of fruit and vegetable by-product fractions is illustrated in Figure 2. Representative tissue issues, priority stabilization and safety checkpoints, and pre-assignment claim boundaries across source/tissue classes are summarized in Table 2.

## 5. Field-to-Postharvest Quality Determinants Before Processing

Before processing, route eligibility is constrained by three upstream questions: which tissue entered the stream, what damage or handling occurred, and whether the material remains sufficiently traceable and stable for further evaluation. Preharvest factors such as cultivar, maturity, production environment, and stress are recorded when they plausibly affect tissue quality. Harvest and sorting records define the organ fraction, damage status, and degree of mixing [9,42,44,45].

After separation, elapsed time, temperature, washing, cutting, packaging, and initial microbial status define the deterioration trajectory [42,43,62,63,64]. We use moisture, water activity, pH, color, odor, visible damage, and microbial indicators as triage variables, though not as complete application specifications. Their role is to identify the dominant instability and determine whether the fraction proceeds to stabilization, requires additional screening, or should be withheld from route assignment.

Fresh-market rejection does not determine route eligibility; the latter depends on tissue identity, safety, stabilization feasibility, and the intended specification.

### Safety, Contaminant, and Regulatory Considerations

Safety screening precedes raw-material candidate status. The required evidence should be selected based on the intended route and the material-specific risk profile. Depending on the fraction, it may address microbial or pathogen risk, pesticide residues, heavy metals, nitrate/nitrite, mold or mycotoxin risk, and relevant physical hazards [65,66,67,68,69,70]. The purpose is not to apply every test to every fraction, but to document hazards that could invalidate the intended route.

Regulatory fit is likewise jurisdiction- and use-specific. In the European Union, a food not used for human consumption to a significant degree within the Union before 15 May 1997 may fall within the scope of Regulation (EU) 2015/2283 [71]; in the United States, an intended food use may require food-additive authorization or a GRAS basis under its intended conditions of use. Regulatory status functions as a decision gate rather than an attribute conferred by plant origin, stabilization, or compositional value. Unresolved safety or legal fit requires further assessment, fallback assignment, or exclusion from route assignment; such cases cannot support a food-grade or food-contact claim.

Accordingly, zero-waste development is a risk-aware route-assignment process rather than a blanket expectation that every by-product re-enter a food or material chain. A fraction becomes a raw-material candidate only after its tissue identity, generation point, postharvest history, microbial status, contaminant profile, stabilization feasibility, and intended application pathway have been sufficiently documented. Screening and specifications should therefore be matched to the intended food, fermentation, coating/film, or selected secondary-material route. Where those requirements remain unresolved, the appropriate outcome is additional assessment, lower-risk assignment, downgrading, or exclusion from route assignment.

Field-to-postharvest records determine whether a fraction proceeds to stabilization and specification or remains on hold pending further evidence. The minimum records, risks associated with missing information, pre-assignment decisions, and readiness outputs across field-to-postharvest stages are summarized in Table 3.

## 6. Stabilization and Preprocessing: Preserving Tissue Quality Before Raw-Material Development

The conversion of fruit and vegetable by-products into raw-material candidates begins before extraction, formulation, or product development. Once plant tissues are detached, trimmed, cut, peeled, pressed, or mixed, they may become more susceptible to quality loss through water migration, respiration, enzymatic browning, pigment degradation, microbial growth, softening, odor development, and compositional instability [42,63,72]. Therefore, stabilization and preprocessing should be understood as tissue-quality preservation steps rather than as merely preliminary operations. In this review, stabilization refers to operations that reduce biological, physicochemical, and microbial instability sufficiently to allow a tissue fraction to be evaluated as a characterized and application-relevant raw-material candidate. This framing is important because fresh-cut and by-product streams often involve tissue disruption, high moisture, and product mixing, which can increase contamination risk and accelerate quality deterioration if sanitation, segregation, temperature control, and drying are not properly managed [42,62,73].

### 6.1. Sorting, Washing, Segregation, and Size Reduction

Stabilization is defined here as the minimum set of operations needed to control the dominant biological, physicochemical, and microbial instability of a defined tissue fraction. The sequence is deliberate: first segregate and remove clearly unsuitable material; next, clean or sanitize as appropriate, and only then select size reduction and stabilization. Washing serves as a risk-reduction step rather than sterilization or evidence of food-grade status [42,62,73].

### 6.2. Blanching, pH Control, and Fermentative Stabilization

Blanching, acidification, and fermentation are selected according to the instability being controlled. Blanching is justified when enzyme activity, microbial load, or drying behavior is the limiting factor; acidification is justified when pH control is required; fermentation is justified when substrate identity and microbial conditions support a reproducible transformation [28,74,75,76,77,78]. No method is assumed to be universally beneficial, and unresolved safety or sensory constraints justify withholding the intended application pathway.

### 6.3. Drying Behavior Across Tissue Types

Drying is selected against the target specification rather than by technological novelty. Tissue density, cell-wall structure, sugar or pectin content, water binding, and surface area influence drying time, shrinkage, browning, stickiness, oxidation, and rehydration [79,80,81,82]. Freeze-drying is therefore retained only as one option when preservation of the structure or thermolabile attributes justifies its cost and scale limitations.

### 6.4. Milling, Powder Properties, and Storage Stability

After drying, particle size, hydration, dispersibility, flow, hygroscopicity, caking, color, odor, and storage stability become specification variables [7,40,83]. A powder is not considered stable because it is dry; stability must be demonstrated under the intended packaging and storage conditions.

### 6.5. Trade-Offs in Stabilization and Preprocessing

**Figure 3:**
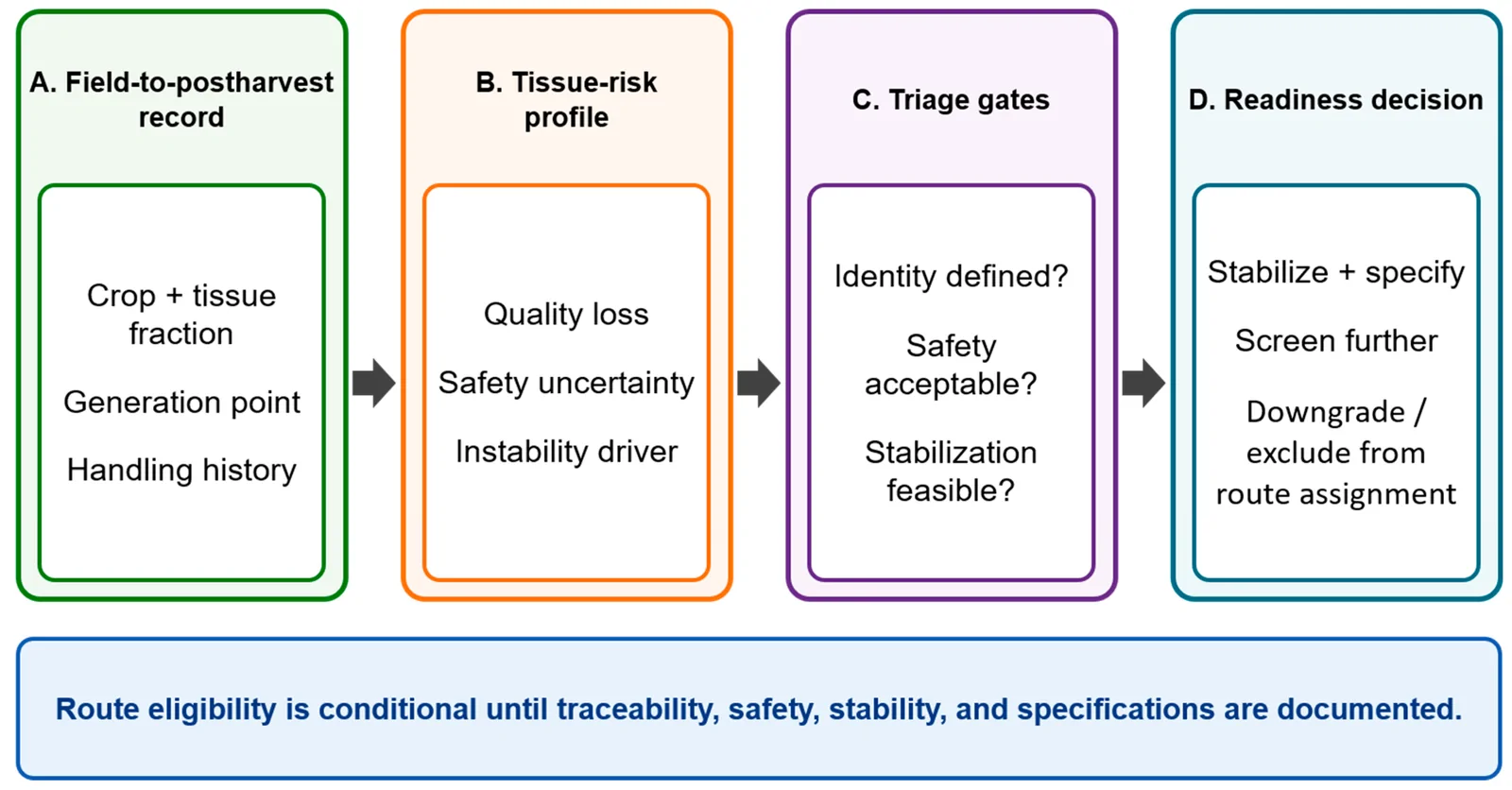
Field-to-postharvest quality triage before stabilization and route assignment. (**A**) Field-to-postharvest records define the crop and tissue fraction, generation point, and handling history before processing. (**B**) These records are interpreted as tissue-risk profiles that identify quality loss, safety uncertainty, and dominant instability drivers. (**C**) The triage gates ask whether identity is defined, safety is acceptable, and stabilization is feasible. (**D**) Fractions then proceed to fit-for-purpose stabilization and specification, additional screening, lower-risk assignment, downgrading, or exclusion from route assignment.

No stabilization method maximizes every relevant attribute. Method selection should therefore prioritize the qualities required by the intended use and document the accepted trade-offs [74,76,77,84]. The field-to-postharvest quality triage process before stabilization and route assignment is illustrated in Figure 3.

**Table 3:** Field-to-postharvest traceability matrix linking minimum records and missing-information risks to decisions made before route assignment and readiness outputs.

Field-to-Postharvest Stage	Minimum Record	Risk If Missing	Decision Before Route Assignment	Readiness Output
**Preharvest context** [10,11,44,45,46]	Species; cultivar or genotype; maturity; season; production context.	Source-related variation may be misattributed to processing, weakening cross-study comparisons.	Confirm crop-level identity and qualify evidence by source and maturity.	Source-identity and maturity record.
**Harvest and segregation** [42,45,72,85]	Harvest date or time; harvest maturity; grading or trimming criteria; mechanical damage; segregation point.	Marketability may be confused with raw-material suitability, and damage effects may be overlooked.	Segregate by organ, tissue, and damage status; defer or exclude poorly defined fractions.	Tissue-fraction, damage, and segregation record.
**Postharvest handling** [42,43,62,63,64,86]	Elapsed time; holding temperature and relative humidity; washing or sanitation; cutting or pressing; packaging or mixing status.	Deterioration may be misattributed to later processing.	Apply controlled holding or rapid stabilization; defer route assignment when the handling history is unclear.	Time–temperature and handling record.
**Fresh tissue quality** [9,10,11,43,86]	Moisture; water activity; pH; color; firmness; odor; visible damage or browning; microbial indicators.	Stabilization may be selected without a defined quality baseline.	Identify the dominant instability and select stabilization or additional screening.	Fresh-quality profile and go/no-go status.
**Safety and contaminants** [65,68,69,70]	Soil or mold exposure; microbial or pathogen indicators; pesticide residues; heavy metals; nitrate/nitrite; mycotoxin risk, as relevant.	Food-grade and other application claims may be unsafe or indefensible.	Defer route assignment, downgrade the intended grade, or exclude the fraction from route assignment until intended-use safety criteria are met.	Safety-screening record and conditional readiness status.
**Stabilization and preprocessing** [28,42,74,81]	Sorting or washing; sanitation; thermal or fermentation conditions; drying endpoint; milling settings.	Process effects and batch reproducibility cannot be interpreted reliably.	Match stabilization to the dominant risk and document critical parameters and process yield where relevant.	Stabilization record and process endpoint.
**Storage before use** [7,10,11,40,83]	Packaging; temperature and relative humidity; light or oxygen exposure; duration; moisture or water activity after storage; color, odor, or marker retention.	Stability claims may overlook caking, oxidation, quality loss, or microbial rebound.	Define a storage window and retest key specifications; downgrade the intended grade or exclude unstable material from route assignment.	Storage-stability profile and validated window.

**Note:** This matrix supports pre-assignment interpretation and does not replace safety or regulatory assessment for the intended use. Readiness outputs indicate conditional evidence status, not automatic food-grade or application eligibility. References are representative rather than exhaustive.

## 7. Composition, Functionality, and Analytical Fingerprinting: From Tissue Markers to Raw-Material Specifications

Composition contributes to specification evidence only when linked to tissue identity, stabilization history, safety, batch variability, and performance in the intended matrix. Primary metabolites, cell-wall polysaccharides, pigments, secondary metabolites, and microstructure can inform material behavior, but no single domain independently determines route eligibility [4,5,8,9].

### 7.1. Primary Metabolites and Nutritional Matrix

Primary metabolites and nutritional components—including carbohydrates, soluble sugars, organic acids, proteins, lipids, minerals, and ash—form the matrix through which tissue-derived functionality is expressed. They influence drying behavior, hygroscopicity, fermentability, pH, color and flavor stability, microbial susceptibility, powder reconstitution, and compatibility with food, fermentation, coating/film, or selected secondary-material routes [4,7,28]. For example, sugar- and acid-rich fruit residues may be compatible with fermentation or flavor-related routes, but after drying they may also show stickiness, browning, caking, or reduced powder flowability when low-molecular-weight sugars, organic acids, and moisture-driven glass-transition behavior are not controlled [83,87,88]. Conversely, seed- or pomace-containing fractions may contribute protein, lipids, dietary fiber, and related nutritional or techno-functional properties, but lipid- or protein-containing matrices may require attention to oxidation, rancidity, emulsifying behavior, and batch variability [89,90].

### 7.2. Cell-Wall Polysaccharides and Dietary Fibers

Cell-wall polysaccharides and dietary fibers provide a key link in tissue-informed raw-material development because they function both as compositional constituents and as structure-forming biopolymers. Pectin, cellulose, hemicellulose, lignin, resistant starch, and soluble or insoluble dietary fiber fractions can influence water-holding capacity, swelling, viscosity, gelation, texture, film formation, fermentability, particle behavior, and storage stability. Plant cell walls are dynamic and complex polymeric structures, and their composition and architecture differ across organs, tissues, developmental stages, and postharvest conditions [8,9].

However, fiber content alone is not sufficient as a raw-material specification. Soluble/insoluble fiber ratio, pectin degree of esterification, uronic acid content, lignification, particle size, microstructure, hydration properties, and interactions with proteins, lipids, starches, and phenolics can help clarify how tissue composition translates into application-relevant functionality.

### 7.3. Pigments and Secondary Metabolites

Pigments and secondary metabolites can serve as useful quality, identity, sensory, and functionality markers for fruit and vegetable by-products. Chlorophylls, carotenoids, anthocyanins, betalains, phenolic acids, flavonoids, tannins, glucosinolates, isothiocyanates, and volatile compounds can contribute to color, antioxidant potential, bitterness, astringency, sulfur notes, aroma, antimicrobial potential, and consumer acceptance [4,91,92,93]. Their distribution can also be tissue- and source-dependent: peels, epidermal tissues, outer leaves, vascular tissues, seeds, and pigmented storage tissues may contain different metabolite profiles even within the same crop.

*Brassicaceae* residues provide a useful case because cabbage, broccoli, cauliflower, radish, napa cabbage, and related crops contain glucosinolates, phenolics, carotenoids, vitamins, minerals, and other phytonutrients. Tissue damage, endogenous myrosinase activity, blanching, drying, fermentation, and thermal processing can substantially alter their sensory and compositional profiles [14,15,76,94].

### 7.4. Tissue-Specific Functional Properties

The functional properties of by-product-derived raw-material candidates arise from the combined effects of tissue structure, particle morphology, hydration state, cell-wall polymers, proteins, lipids, minerals, and phytochemicals within a specific processing or application matrix [7,95,96]. Relevant techno-functional properties include water-holding capacity, oil-holding capacity, swelling capacity, solubility, wettability, dispersibility, viscosity, gelation, emulsification, foaming, film-forming ability, fermentability, and reconstitution behavior. These properties are most informative when they are linked to the intended route, because the same tissue fraction may function differently as a fiber-rich powder, fermentation substrate, coating or film-forming fraction, or selected material-route candidate.

We therefore prioritize evidence that connects composition to measurable properties such as hydration, particle behavior, texture, gelation, fermentability, film formation, sensory quality, or mechanical compatibility. Total phenolic or flavonoid contents and radical-scavenging assays are screening indicators that provide screening-level evidence; compound-specific and performance claims require targeted identification and application-level validation [40,95,97,98].

### 7.5. Analytical and Fingerprinting Methods

Analytical methods were evaluated in relation to the claims they were intended to support. Moisture, water activity, pH, color, particle size, microbial status, and contaminants provide baseline quality and safety evidence. Targeted chromatography or mass spectrometry is appropriate when specific compound claims are made; spectroscopic or untargeted fingerprints are more useful for batch comparison and screening; microscopy is useful when structure–function relationships are central [4,8,24,99]. No single platform is proposed as universally necessary.

A defensible specification links method selection, tissue identity, stabilization history, marker evidence, safety status, batch variability, and performance in the intended application. Study-specific evidence for apple pomace, red-radish leaf and root fractions, carrot peels, and cabbage outer-leaf and leaf/core fractions is summarized in Supplementary Table S1, together with the reported analytical bases, evidence-use classifications, and principal limitations [100,101,102,103,104]. The minimum analytical evidence and corresponding claim boundaries for the principal specification domains are summarized in Table 4.

These specification domains form the evidence gates applied before route assignment.

## 8. Tissue-to-Raw-Material Framework for Zero-Waste Raw-Material Development

**Figure 4:**
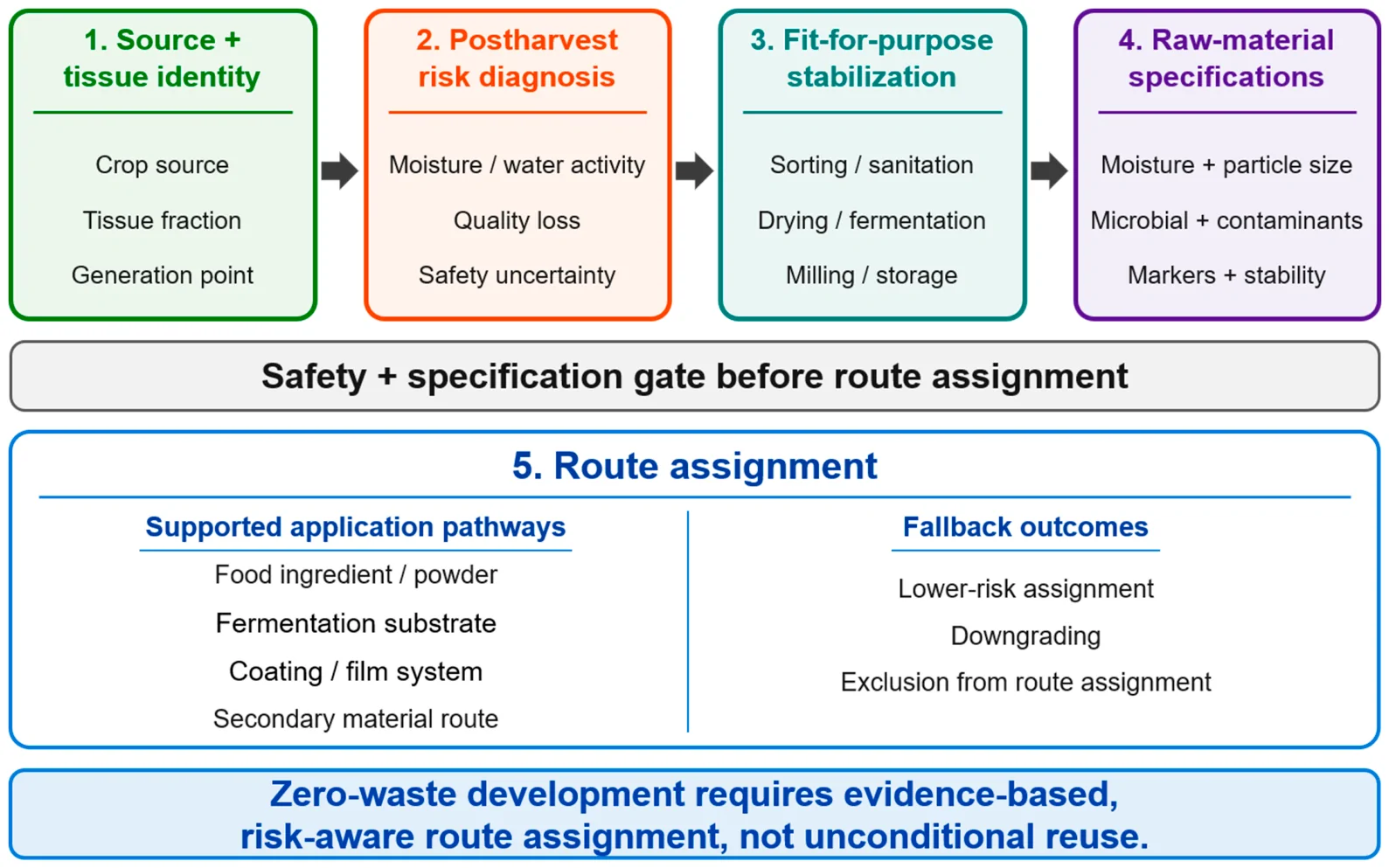
Tissue-to-raw-material framework for zero-waste development of fruit and vegetable by-product fractions. The framework links source and tissue identity, postharvest-risk diagnosis, fit-for-purpose stabilization, raw-material specifications, and route assignment. The safety and specification gate precedes route assignment. Supported application pathways require evidence matched to the intended use; lower-risk assignment, downgrading, or exclusion from route assignment remains valid when traceability, safety, stability, or specification evidence is insufficient.

The framework links five decisions: material identification, postharvest-risk diagnosis, fit-for-purpose stabilization, specification setting, and route assignment. An application pathway is supported only when identity, safety, stability, and the relevant intended-use specifications are documented. The five-step tissue-to-raw-material framework and the safety and specification gate preceding route assignment are illustrated in Figure 4.

### 8.1. Step 1: Identify Crop Source and Tissue Fraction

Step 1 records the crop, organ or tissue fraction, generation point, maturity or production context, where relevant, and segregation status. This record is the identity anchor for all subsequent interpretation; poorly defined mixed streams do not proceed directly to tissue-specific or application-specific claims.

### 8.2. Step 2: Diagnose Postharvest Risk

Step 2 identifies the dominant risk from handling history, water status, tissue injury, sensory deterioration, and microbial or contaminant uncertainty. The output is a tissue-specific risk profile—not a generic label such as “perishable”—that determines whether immediate stabilization or additional screening is required.

### 8.3. Step 3: Select Stabilization Route

Step 3 selects the minimum stabilization needed to control the dominant risk while preserving the attributes required by the intended route. Methods are not ranked universally; the selected operation must be justified against the target specification and its documented trade-offs.

### 8.4. Step 4: Define Raw-Material Specifications

Step 4 defines only the specifications that govern the intended route, including identity, moisture or water activity, particle or structural properties, safety criteria, relevant markers, and storage stability. Regulatory fit is included when it affects route eligibility. Until these criteria are met, the processed fraction remains a raw-material candidate supported only by screening-level or specification-supporting evidence.

### 8.5. Step 5: Assign the Tissue-Derived Candidate to an Application Pathway

Step 5 selects either a supported application pathway or a fallback outcome. Food ingredients or powders, fermentation substrates, coating/film systems, and selected secondary-material pathways require application-level validation; lower-risk assignment, downgrading, or exclusion from route assignment applies when entry-gate or validation evidence remains insufficient.

### 8.6. Framework Output and Practical Use

The framework output is a traceable decision record linking material identity, risk profile, stabilization, specifications, and route outcome. For researchers, it defines the evidence needed before application claims are made; for industry users, it supports incoming-batch triage and identifies where additional screening, reclassification, or exclusion is required.

## 9. Application Pathways: Specification-Based Route Assignment for Plant-Based Food Development and Selected Secondary Uses

Four application pathways were evaluated against the same decision criteria: candidate fit, minimum entry gate, application-level evidence, and fallback outcome. These pathways comprise whole-tissue powders, fermentation substrates, coating or film systems, and selected secondary-material uses.

### 9.1. Whole-Tissue Powders as Functional Ingredients

Whole-tissue powders are most defensible when the combined tissue matrix—rather than one isolated compound—is intended to provide fiber, color, flavor, minerals, hydration, or particle-level functionality [7,25,26,88]. Their use requires acceptable particle behavior, sensory quality, microbial and contaminant status, and storage stability in the target matrix. Claims should remain nutritional or techno-functional unless stronger application or health evidence is available.

### 9.2. Fermentation Substrates

Fermentation is retained when substrate identity, composition, microbial context, and process endpoint can be controlled [2,28,75,78]. It is not a default solution for wet residues. A supported route requires a documented pH trajectory, microbial safety, sensory outcome, and storage endpoint, with additional attention to sulfur and bitterness in Brassicaceae tissues.

### 9.3. Edible Coatings and Films

Coating or film routes are considered when the polymer identity and film-forming behavior are demonstrated [33,34,35]. Antioxidant or antimicrobial screening alone is insufficient; mechanical and barrier properties, adhesion, migration or food-contact safety, sensory effects, and storage stability must be validated for the intended system.

### 9.4. Selected Secondary Material Routes Based on Plant Tissue Traits

Secondary-material routes are considered when structural fibers, particle networks, absorbency, or biopolymer behavior provide measurable functionality [109,110,111]. Their inclusion does not imply automatic sustainability: processing feasibility, realistic input variability, safety, cost, and end-of-life performance remain part of the route decision.

### 9.5. Application Constraints

Color, odor, bitterness, particle behavior, microbial or contaminant risk, batch variability, and regulatory uncertainty function as route-assignment variables rather than secondary inconveniences [107,112,113,114]. An application pathway is supported only when its minimum entry gate and validation evidence are met; otherwise, the fraction moves to lower-risk assignment, downgrading, or exclusion from route assignment. Candidate fit, minimum gates, application-level validation requirements, and claim boundaries or fallback decisions for each route are summarized in Table 5.

## 10. Knowledge Gaps and Critical Discussion

Six recurring evidence gaps constrain cross-study comparison and defensible route assignment: terminology, tissue identity, postharvest history, analytical depth, specification and regulatory reporting, and scale-up. Together, these gaps define the minimum reporting floor summarized in Table 6.

### 10.1. Terminology Gap

Terminology is operational, not merely semantic. Studies should state whether a material is described as waste, residue, side-stream, potential by-product, raw-material candidate, specified raw material, or application-validated input, because these terms represent different levels of control and claim readiness [3,37,38]. Removal from the primary product stream does not by itself determine waste status or eligibility for a new application pathway.

### 10.2. Tissue Reporting Gap

Crop name alone is insufficient to identify the starting material. Organ, tissue fraction, maturity or cultivar (where relevant), generation point, damage status, and mixing or segregation should be reported because they can explain variation in water status, structure, phytochemical localization, microbial exposure, and processability [4,14,15,120,121].

### 10.3. Postharvest History Gap

Postharvest history is needed to distinguish source effects from treatment effects. At a minimum, studies should report elapsed time, temperature, washing or sanitation, cutting or damage status, storage conditions, and the interval to stabilization; otherwise, changes in composition, color, powder behavior, or microbial status cannot be attributed confidently to the evaluated process [9,10,11,122].

### 10.4. Analytical Depth Gap

Total phenolic or flavonoid contents and antioxidant assays are screening indicators that provide preliminary evidence but do not identify compound classes, tissue localization, degradation products, hazards, sensory-active compounds, or performance in the intended application [5,24,97,98]. Analytical depth should therefore be selected based on the intended claim: targeted evidence for compound claims, structural evidence for functionality claims, and application-level testing for performance claims.

### 10.5. Standardization and Regulatory-Reporting Gap

A process demonstration alone is insufficient to define a reusable raw-material specification. Studies should define intended-use quality and safety criteria and report the jurisdiction and intended use when regulatory status is relevant [66,67,123]. EU novel-food status and U.S. food-additive or GRAS pathways illustrate why plant origin and stabilization alone cannot establish legal eligibility; an exhaustive comparison of national systems is outside the scope of this review.

### 10.6. Scale-Up Gap

Scale-up evidence should address realistic input variability, supply continuity, segregation logistics, stabilization yield, mass balance, energy and water use, storage, safety, sensory acceptance, regulatory pathways, and techno-economic feasibility [2,13,124,125,126,127,128]. Laboratory conversion demonstrates technical possibility, not industrial or environmental viability.

Together, these gaps define a minimum reporting floor rather than a universal pass/fail standard. Supplementary Table S2 illustrates how reporting completeness constrains the interpretation of each domain.

This review has several methodological limitations. Google Scholar was used as a supplementary web-search source and was limited a priori to the first 50 accessible relevance-ranked results for each of five predefined queries. The numbers inspected and captured at the retrieval stage were 50, 50, 50, 43, and 34. These records then entered the common deduplication and title/abstract eligibility-screening workflow. Backward and forward citation searching were not performed. H.J.W. conducted the primary title/abstract screening and full-text assessment, and A.-j.C. verified the recorded screening and full-text eligibility outcomes. Heterogeneity in tissue definitions, material conditions, analytical methods, units, and application contexts precluded quantitative pooling. These limitations were addressed through source-specific search reporting, a retained record-level audit trail, study-specific quantitative presentation, and claim boundaries matched to the evidence level.

## 11. Future Perspectives for Tissue-Informed Raw-Material Systems

Future work should prioritize three linked tasks: defining intended-use quality targets before processing, classifying tissue fractions rapidly and reproducibly, and maintaining interoperable records that connect source, stabilization, specification, and outcomes.

### 11.1. Quality-by-Design as a Planning Framework

Quality-by-design is used here as a planning analogy, not as a direct regulatory transfer from pharmaceutical development. The practical sequence is to define the intended raw-material profile, identify the tissue attributes and process parameters that control it, and establish monitoring and acceptance criteria [124,129,130,131]. For tissue-derived fractions, this approach shifts attention from generic compositional richness to predefined material performance, documented trade-offs, and evidence matched to the intended application.

### 11.2. Decision-Oriented Imaging and Tissue Classification

Imaging and nondestructive sensing are most useful when they improve a defined decision—such as tissue classification, damage detection, freshness assessment, or batch segregation—and are validated against reference measurements [86,99,132]. For by-product fractions, their main value lies in rapid triage before stabilization and in linking structural features to drying, milling, hydration, or route performance. Stand-alone images without calibration, decision thresholds, or independent validation should not be interpreted as specification evidence.

### 11.3. Interoperable Digital Raw-Material Specifications

Digital specifications are useful only when they integrate tissue identity, postharvest history, stabilization, safety, analytical evidence, and route outcome rather than logistics alone [120,121,133]. Metadata principles exemplified by the Minimum Information About a Plant Phenotyping Experiment (MIAPPE) could support batch comparison, interoperability, and traceability across studies and supply chains. Digitalization cannot compensate for missing source data or poorly defined specifications; it should make evidence auditable, not merely electronic.

### 11.4. Brassicaceae as a Comparative Case Study Platform

*Brassicaceae* provide a useful comparative platform because related crops generate contrasting leafy, root, stem, core, stalk, and trimming tissues within a shared botanical background [6,14,15,57,134]. Future studies should test whether tissue-level records predict stabilization response and route performance across crops, rather than compile additional crop-wide lists of glucosinolates or phenolics. Napa cabbage and radish remain regionally relevant test materials, but the inference should remain tissue-based rather than geography-specific. This does not imply breeding crops for waste; it asks whether predictable side-stream quality can be considered alongside primary-product and postharvest quality [45,100].

### 11.5. Preventing Avoidable Quality Loss as the Operational Objective

We propose preventing avoidable quality loss as the operational objective underlying zero-waste development. The first decision is whether a tissue fraction can be identified and preserved before deterioration; the second is whether the resulting evidence supports a safe, stable, and reproducible route [9,43,86,122]. This framing retains circularity while avoiding the implication that every residue should enter a high-value application.

## 12. Conclusions

This review reframes fruit and vegetable by-products as postharvest tissue fractions whose route eligibility depends on documented identity, quality history, stabilization, safety, and intended-use specifications. Tissue segregation and field-to-postharvest records establish the basis for fit-for-purpose stabilization, whereas composition and analytical fingerprints become decision-relevant only when linked to measurable raw-material properties and application-level performance. The framework defines zero-waste development as evidence-based route assignment, including lower-risk assignment, downgrading, or exclusion where traceability, safety, stability, or specification evidence remains insufficient. Future progress will depend more on tissue-level reporting, storage validation, safety and regulatory documentation, scale-up evidence, and reproducible performance under defined application conditions than on expanding lists of potential uses.

## Figures and Tables

**Table 1:** Positioning of the present tissue-informed review relative to representative fruit and vegetable by-product review streams.

Representative Review Stream	Typical Organizing Focus	Tissue-Informed Extension in This Review
**Green extraction and compound recovery** [5,20,21,22,23]	Extraction solvents and enabling technologies; process efficiency; extraction yield; target-compound recovery.	Defines crop source, organ/tissue fraction, postharvest condition, and stabilization status before evaluating recovery potential.
**Chemical profiling and bioactivity** [4,5,24]	Compound classes; analytical platforms and profiles; antioxidant or antimicrobial screening; compositional richness.	Distinguishes screening-level evidence from raw-material specifications and links composition to tissue identity, safety, stability, and validation in the intended application.
**Biorefinery and circular economy** [2,12,13]	Cascade use; multi-output valorization; resource recovery; circular bioeconomy and supply-chain logic.	Adds tissue segregation, postharvest quality, safety, and specification gates to system-level route assignment.
**Food formulation and functional ingredients** [4,25,26,27]	Food matrices; fortification level; sensory, nutritional, and techno-functional performance.	Places tissue diagnosis, stabilization history, batch variability, and route eligibility upstream of formulation.
**Fermentation-based by-product use** [28,29,30,31]	Substrate conversion; starter or native microbiota; pH trajectory; fermentation performance.	Treats fermentation as a tissue- and process-specific route requiring defined substrate identity, safety status, and endpoint control.
**Coating, film, and selected material routes** [32,33,34,35]	Coating or film formulation; barrier or active performance; biopolymer behavior; material testing.	Links tissue-derived polymers and markers to source identity, stabilization history, food-contact relevance, and performance validation in the intended system.
**Crop-specific by-product reviews** [6,14,15,36]	Single-crop or commodity-specific composition, bioactives, processing routes, and potential applications.	Uses Brassicaceae as a representative case study while integrating organ-specific evidence into a cross-crop tissue-to-raw-material framework.

**Note:** Review streams are representative and may overlap. The table outlines the scope and organizing logic of the present review rather than ranking or criticizing the prior literature. References are representative rather than exhaustive.

**Table 2:** Tissue-specific screening matrix linking representative source/tissue classes with dominant tissue issues, priority stabilization/safety checkpoints, and claim boundaries before route assignment.

Source/Tissue Class	Dominant Tissue Issue	Priority Stabilization/ Safety Checkpoint	Claim Boundary Before Route Assignment	References
**Fruit peels** **(citrus, apple, mango, berry, tomato)**	Protective or epidermal tissue; often rich in pectin, fiber, pigments, or phenolics; high surface exposure.	Confirm source and peel separation; document the postharvest interval; control browning, microbes, drying sensitivity, pigment loss, and surface contaminants.	Application suitability requires documented stabilization, contaminant control, and validation in the intended application in addition to compositional evidence.	[4,49,50,51]
**Fruit pomace and pulp residues** **(apple, grape, berry, tomato pomace)**	Wet peel–pulp–seed matrix; variable fraction ratio; stickiness, caking, browning, and batch variability.	Record crop and process, peel–pulp–seed ratio, water activity, microbial status, drying or fermentation history, and storage stability.	Pomace is not a uniform material; claims must be qualified by fraction ratio, stabilization history, microbial control, and storage conditions.	[2,25,26,52]
**Seeds and press cakes** **(fruit seeds, seed residues, pressed cakes)**	Dense storage tissue; lipid-, protein-, fiber-, or mineral-rich; oxidation, hard structure, and allergen or antinutritional concerns.	Assess oxidation or rancidity, milling behavior, allergens, antinutritional factors, sensory constraints, contaminant status, and batch variability.	Food or material use requires safety, sensory, processing, and regulatory evidence matched to the intended application in addition to compositional evidence.	[53,54,55,56]
**Vegetable outer leaves** **(napa cabbage, cabbage, broccoli or cauliflower leaves)**	High-moisture exposed leafy tissue; fragile and pigment- or fiber-rich; rapid deterioration and surface-contamination risk.	Document time–temperature history; apply washing or sanitation and rapid stabilization; assess microbial status, pesticides, nitrate or nitrite, and other relevant contaminants.	Leaf-derived claims require documented tissue identity, rapid stabilization, safety screening, sensory assessment, and later route validation.	[6,14,57,58]
**Vegetable stems, stalks, petioles, and cores** **(broccoli stalks, cabbage cores, cauliflower stems, petioles)**	Fibrous structural tissue; possible lignification, coarse texture, variable particle behavior, and odor or bitterness constraints.	Define size reduction and drying or milling behavior; characterize fiber profile, particle properties, texture or mouthfeel, odor or bitterness, and safety.	Suitability depends on sensory, texture, particle-behavior, safety, and application-performance evidence rather than on fiber content alone.	[4,6,14,59]
**Roots, root peels, and root trimmings** **(radish, carrot, parsnip peels or trimmings)**	High-water storage or root tissue with soil-contact surfaces; variable sulfur notes, color, texture, and contaminant exposure.	Document delay and washing or soil removal; assess microbes, pesticides, heavy metals, nitrate or nitrite where relevant, and postharvest condition.	Later claims depend on documented soil-contact risk, segregation, contaminant screening, stabilization history, and tissue-specific sensory constraints.	[48,59,60,61]
**Brassicaceae leafy, root, and stem residues** **(napa cabbage, radish, cabbage, broccoli, cauliflower, kale)**	Marked organ heterogeneity; glucosinolate–myrosinase changes, sulfur or bitterness, pigment loss, and moisture vary by tissue.	Report organ and tissue fraction, damage, myrosinase-related state, odor or bitterness, stabilization history, safety profile, and relevant markers.	Value and suitability require organ-specific stabilization, sensory, safety, and application evidence; glucosinolate content is only one supporting measure.	[6,14,15,44]
**Mixed or poorly segregated residues** **(unsegregated trimming or sorting residues; mixed peel–leaf–stem–root streams)**	Heterogeneous composition, moisture, structure, microbes, and odor; tissue identity and traceability are reduced.	Re-segregate where feasible; classify hazards; assess water activity, microbial and contaminant status, drying uniformity, and traceability.	Without tissue identity, segregation, safety screening, and specifications, specific food-grade, bioactive, or ingredient claims remain unsupported; exclusion from route assignment is appropriate.	[2,4,13,42]

**Note:** Source/tissue classes and references are representative rather than exhaustive. The listed checkpoints define tissue-specific screening priorities and pre-assignment claim boundaries; they do not constitute complete intended-use specifications.

**Table 4:** Specification matrix defining minimum analytical evidence and claim boundaries for raw-material candidates from fruit and vegetable by-products.

Specification Domain	Representative Tissue Context	Minimum Analytical Evidence	Interpretation and Claim Boundary
**Moisture and water activity** [7,9,10,11,65]	High-moisture leafy fractions, pomace, root trimmings, fresh-cut residues, and stabilized powders.	Moisture and water activity; packaging and storage conditions; sorption or stability testing when storage claims are made.	Stability should be interpreted jointly from moisture, water activity, microbial status, packaging, and storage history.
**Primary metabolites and nutritional matrix** [4,5,24,28,87,88,89,90,105]	Pulp, pomace, roots, seeds, press cakes, and storage tissues.	Proximate composition; sugar and organic-acid profiles; ash and minerals; pH where relevant.	Candidate status additionally requires documented tissue fraction, maturity, generation point, and stabilization history.
**Cell-wall polysaccharides and dietary fiber** [4,7,8,9,40,95,106]	Peels, pomace, stems, stalks, outer leaves, roots, and fibrous trimmings.	Soluble and insoluble fiber; pectin or other cell-wall fractions; particle size; hydration or rheological testing where relevant.	Application-relevant functionality must be supported by evidence for texture, dispersibility, mouthfeel, gel or film behavior, or matrix performance in addition to fiber or pectin content.
**Pigments and color markers** [4,5,15,76,91,92,93]	Peels, outer leaves, epidermal tissues, pigmented pulp, and other colored plant tissues.	Colorimetry; targeted pigment analysis when pigment claims are made; retention during stabilization and storage.	Pigment identity and stability require analytical confirmation together with oxygen, light, pH, temperature, and storage context.
**Phenolics, flavonoids, and antioxidant-screening markers** [4,5,24,97,98]	Peels, seeds, pomace, outer tissues, and selected Brassicaceae fractions.	Total phenolic and flavonoid contents as screening metrics; targeted profiling when compound claims are made; assay conditions, concentration, and test matrix.	Radical-scavenging assays are screening indicators that provide screening-level evidence; antioxidant or antimicrobial claims require targeted and application-level validation.
**Glucosinolate, sulfur, and odor-active markers** [14,15,44,76,94,107]	Brassicaceae leaves, roots, stems, trimmings, damaged tissues, and fermented substrates.	Targeted glucosinolate and isothiocyanate profiles; myrosinase-related state; volatile or sensory assessment where relevant.	Tissue damage, blanching, drying, fermentation, and leaching can alter these profiles; suitability cannot be inferred from glucosinolate content alone.
**Microstructure and particle morphology** [7,8,9,40,99]	Dried or milled tissues, powders, films, gels, fiber-rich fractions, and rehydrated matrices.	Particle-size distribution; microscopy or image analysis where needed; texture, rheology, hydration, or reconstitution tests.	Descriptive images alone are insufficient; structural evidence must be linked to measurable functional performance.
**Safety and contaminant markers** [62,65,66,67,68,69,70,108]	Soil-contact roots, leafy tissues, fresh-cut residues, pomace, damaged or mold-prone tissues, and mixed residues.	Microbial indicators and pathogen-risk assessment; pesticide residues; heavy metals; nitrate/nitrite; mycotoxins or physical hazards, as relevant.	Safety and regulatory fit are prerequisites for application claims; unresolved hazards preclude application-ready status and require further screening, downgrading, or exclusion from route assignment.

**Note:** Specification domains should be selected according to tissue fraction, stabilization history, intended route, and validation level; they are not mandatory tests for every fraction. Supplementary Table S1 provides study-specific quantitative examples, analytical bases, evidence-use classifications, and principal limitations. References are representative rather than exhaustive.

**Table 5:** Route-assignment matrix linking tissue-derived raw-material candidates with minimum gates, application-level validation, and fallback decisions.

Route or Decision	Candidate Fit	Minimum Gate	Application-Level Validation	Claim Boundary or Fallback
**A. Supported application routes**				
**Food ingredient or whole-tissue powder** [4,7,25,26,27,88,115]	Stabilized, traceable pomace, peel, leafy, root, or Brassicaceae fractions with acceptable fiber, color, hydration, sensory, and particle characteristics.	Defined identity and stabilization history; moisture and water activity; particle size; sensory profile; microbial and contaminant status; storage stability.	Hydration or reconstitution; texture and mouthfeel; performance in the target food matrix; sensory acceptance; microbial and storage stability.	Ingredient or techno-functional claims require matrix-level evidence. Redirect the fraction if grittiness, bitterness, sulfur notes, caking, color instability, microbial risk, or unsupported health claims remain.
**Fermentation substrate** [2,28,29,30,75,78,116]	Defined pomace, leafy, root, or mixed fractions with fermentable substrate characteristics and a controllable microbial context.	Substrate identity and consistency; pH and sugar profile; microbial and contaminant status; time–temperature history; salt tolerance where relevant.	Controlled fermentation; pH trajectory; starter or native-microbiota performance; microbial safety; sensory outcome; storage stability.	Fermentation is not a default solution for wet residues. Redirect or exclude fractions when spoilage, pathogen risk, off-odor, sulfur or bitterness, or inconsistent endpoints remain unresolved.
**Coating or film system** [32,33,34,35,117,118]	Pectin-, polysaccharide-, phenolic-, or pigment-containing fractions with defined film-forming or active-preservation potential.	Polymer or fiber identity; film-forming or rheological behavior; relevant marker compounds; microbial and contaminant status; solvent or residue limits; food-contact relevance where intended.	Film formation; mechanical and barrier properties; adhesion; migration or food-contact safety; active performance; sensory and storage stability.	Screening-level antioxidant or antimicrobial evidence alone is insufficient for application-performance claims. Redirect fractions when the system is weak, highly hydrophilic, unstable, sensory-limiting, or legally unsupported.
**Secondary material route** [32,33,109,110,111,119]	Fiber-rich or structurally robust fractions with defined reinforcement, absorbency, particle-network, or biopolymer potential.	Fiber and particle profile; moisture; contaminant status; fractionation or modification history; mechanical compatibility.	Mechanical or barrier performance; processing feasibility; stability under realistic input variability; cost or energy context; end-of-life behavior.	Material claims require performance and feasibility evidence for the selected application pathway. Redirect fractions when variability, processing burden, end-of-life uncertainty, or safety or regulatory gaps remain.
**B. Fallback decisions for unmet intended-use criteria**				
**Lower-risk assignment** [2,13,107,108,112]	Traceable fractions that do not meet one or more requirements of the intended route but retain a documented hazard status compatible with a defined lower-risk route.	Defined hazard and contaminant status; residual-risk assessment; stability; intended fallback route; handling and storage requirements.	Technical, environmental, safety, and regulatory fit for the specified lower-risk route under realistic input variability.	Lower-risk assignment is a valid decision, not a failed zero-waste outcome. Claims associated with the original higher-requirement application pathway must not be retained.
**Downgrading** [2,13,107,108,112]	Fractions that remain usable but fail one or more quality, sensory, stability, or specification criteria for the intended grade or application.	Document the unmet criterion and define the revised material grade, intended use, and corresponding safety and handling requirements.	Confirm suitability and stability under the downgraded specification or less demanding application conditions.	Downgrading requires explicit reclassification; the original food-grade, functional, active, or high-value claim must not be carried forward.
**Exclusion from route assignment** [13,107,108,112]	Spoiled, moldy, contaminated, untraceable, legally restricted, or highly variable fractions that cannot meet minimum requirements.	Document the unmet traceability, safety, stability, legal, or specification criterion and the basis for exclusion from route assignment.	No application validation is pursued; document the exclusion decision and the applicable regulated management pathway.	Exclusion from route assignment preserves claim credibility. Composting, anaerobic digestion, or another regulated management option may be more appropriate where permitted.

**Note:** This matrix supports route assignment after traceability, stabilization, safety screening, and specification setting; it does not replace safety or regulatory assessment for the selected application. Supported pathways require application-level validation, whereas lower-risk assignment, downgrading, and exclusion from route assignment remain valid for fractions that fail one or more intended-use criteria.

**Table 6:** Minimum reporting checklist for tissue-informed fruit and vegetable by-product raw-material studies.

Reporting Domain	Minimum Reporting Requirement	Claim-Control Function
**Material status and intended route**	State the material status (e.g., waste, residue, by-product, side-stream, stabilized raw-material candidate, specified raw material, preliminary ingredient candidate, or application-validated input).	Prevents legal, food-grade, and value-chain overclaiming by clarifying material status and intended use.
**Crop, organ, and tissue identity**	Report crop species and common name; cultivar or genotype when available; organ and tissue fraction; relevant anatomical identity; and segregation or mixing status.	Links tissue structure and compositional variability to stabilization, specification, and route decisions.
**Generation point and quality history**	Report the generation point; harvest or processing date; sorting or trimming criteria; damage status; elapsed time to stabilization; storage and packaging conditions; and washing or sanitation history.	Prevents deterioration or compositional change from being misattributed to downstream processing.
**Fresh quality and safety screening**	Report moisture, water activity, pH, color and odor, visible damage, microbial indicators, and route-relevant contaminants (e.g., pesticide residues, heavy metals, nitrate/nitrite, or mycotoxin risk), as applicable to the tissue and intended route.	Supports safe route assignment and documents criteria for additional screening, downgrading, or exclusion from route assignment.
**Stabilization, preprocessing, and storage**	Report the applied sorting, washing or sanitation, blanching, drying, fermentation or acidification, milling, packaging, storage conditions, and defined stability window.	Supports reproducibility, batch comparison, storage validation, and specification setting.
**Analytical evidence and specifications**	Report claim-relevant compositional markers or domains; fiber or polysaccharide profiles where relevant; particle-size or structural evidence; microbial and contaminant criteria; and final raw-material specifications.	Distinguishes screening evidence from raw-material specifications and limits unsupported application claims.
**Application validation and route decision**	Report the target matrix or application pathway; performance under the tested conditions; sensory compatibility; safety and regulatory fit; scale-up considerations; and criteria for lower-risk assignment, downgrading, or exclusion from route assignment.	Links claims to actual use conditions and documents alternative route decisions.
**Regulatory status and intended-use legal fit**	Report the jurisdiction and intended route; history of food use where relevant; regulatory status or uncertainty; authorization or notification requirements; food-contact and migration relevance; and labeling or conditions-of-use constraints.	Prevents the assumption that plant origin, stabilization, or compositional value automatically confers food-grade, food-contact, or cross-jurisdictional regulatory eligibility.

**Note:** This checklist is a reporting aid and does not replace safety and regulatory assessment for the intended use, life-cycle assessment, or techno-economic analysis. Supplementary Table S2 provides author-constructed paired examples of ambiguous or insufficient and interpretation-ready reporting. These examples illustrate how reporting completeness constrains evidence interpretation and are not universal pass/fail criteria.

## Data Availability

No primary experimental data were generated. Complete source-specific search strategies, search dates, retrieval counts, process chronology, record-management procedures, and citation-selection criteria are provided in Supplementary Table S3, and the evidence-flow counts and full-text exclusion summary are provided in Supplementary Figure S1. The evidence bank contained 872 eligible reports. The 134-item manuscript reference list served a separate citation function and included eligible reports cited directly for specific claims or synthesis domains, together with contextual, methodological, regulatory, and reporting-policy sources, which were used where needed.

## Supplementary Materials

The following supporting information can be downloaded at: https://www.mdpi.com/article/10.3390/plants15162423/s1. Supplementary Figure S1: Evidence-flow summary of the literature identification, screening, and eligibility process. Supplementary Table S1: Selected study-specific quantitative evidence linking tissue identity, postharvest state, stabilization, composition, and application-matrix performance. Supplementary Table S2: Author-constructed paired examples showing how reporting completeness constrains evidence interpretation across the tissue-to-raw-material framework. Supplementary Table S3: Source-specific search strategies, search chronology, retrieval counts, record-management procedures, and manuscript citation-selection criteria used in the integrative evidence-mapping review.

## Abbreviations

GRAS	generally recognized as safe
