# Supplementary material for: Fruit and Vegetable By-Products as Postharvest Tissue Fractions: A Raw-Material Framework for Plant-Based Food Development
Source: Plants (Basel). 2026 Aug 8;15(16):2423. doi: 10.3390/plants15162423 (PMC13516057; doi:10.3390/plants15162423)
Supplement: Supplementary file 1 [file plants-15-02423-s001.zip › plants-4407687_Supplementary_Table_S1.pdf]

Supplementary Table S1. Selected study-specific quantitative evidence linking tissue identity, postharvest state, stabilization, composition, and application-matrix performance.

Scope and interpretation. Values are reproduced in the units, analytical bases, and numerical precision reported by the primary studies, except where a study-specific source note explains the selected value or unit; non-equivalent materials, methods, and moisture bases were not pooled. The studies were selected as quantitative exemplars across tissue identity, stabilization, and application contexts rather than as an exhaustive inventory. In this table, "specification-supporting" denotes evidence that may inform candidate attributes, ranges, or process conditions; it does not constitute a validated material specification or release criterion.

Reporting codes and common boundary. NR, not reported in the cited article or its Supplementary Materials; NE, not evaluated in the cited study; NA, not applicable to the study design. Unless stated otherwise, the cited studies did not establish independent source-lot replication or industrial production-batch validation and therefore do not demonstrate lot-to-lot reproducibility, validated storage windows, or commercial release specifications.

Panel A. Tissue-defined compositional and within-study comparator evidence

Reference and exact source locator	Crop and tissue-defined material	Postharvest state and replication	Stabilization or analytical condition	Quantitative endpoint and unit/basis	Evidence role in this review	Main claim boundary
[101] Sudha et al. (2007) DOI: 10.1016/j.foodchem.2006.12.016 Composition: Table 1 (p. 688); phenols: Table 5 (p. 691).	Dried apple pomace from a fruit-juice processor, consisting of peel, stem, seed, and residual material from juice extraction; ground to pass a 150 µm sieve.	Dried pomace was obtained from one identified fruit-juice processor; the number of independent source lots and the elapsed post-production history were NR. Chemical analyses were performed in triplicate.	Dried, finely ground pomace was chemically characterized; no validated post-drying storage window was evaluated.	Moisture 10.80 ± 0.03%. Ash 0.50 ± 0.05%, fat 2.70 ± 0.10%, and protein 2.06 ± 0.05% (14% moisture basis). TDF 51.10 ± 1.86%, IDF 36.50 ± 1.14%, and SDF 14.60 ± 0.14% (dry basis; mean ± SD, n = 3). Total phenols 10.16 mg GAE/g[a] (water-extractable, 4.65; methanol-extractable, 5.51 mg GAE/g).	Specification-supporting compositional evidence for a defined pomace fraction.	Single industrial-source context. Cultivar and independent lot variability were NR; microbial/contaminant status, water activity, and storage stability were NE.
[102] Goyeneche et al. (2015) DOI: 10.1016/j.jff.2015.04.049 Tables 1-4 (pp. 258-261).	Red radish (*Raphanus sativus* L.) leaves as the candidate leafy fraction; roots as a within-study edible-organ comparator, not as a by-product fraction.	Field-grown radishes purchased from a local market were held at 5 ± 1 °C in darkness before processing. Roots and leaves were separated, washed, and analyzed fresh immediately after washing. The duration of chilled holding and the number of independent source lots were NR; measurements were performed in triplicate.	No stabilization process or application matrix was evaluated. Only chilled holding before fresh-tissue analysis was described; physicochemical, nutritional, microbiological, and antioxidant properties were characterized.	Leaves vs roots, on a fresh-weight basis unless otherwise stated: moisture 89.50 ± 0.98 vs 95.24 ± 0.29 g/100 g FW; protein 3.81 ± 0.32 vs 0.57 ± 0.09 g/100 g FW; ash 1.70 ± 0.06 vs 0.77 ± 0.07 g/100 g FW. Ca 752.64 ± 1.45 vs 147.87 ± 1.31 mg/100 g FW[b]; ascorbic acid 38.69 ± 1.99 vs 16.59 ± 0.29 mg/100 g FW. TPC 695.07 ± 36.67 vs 341.45 ± 5.70 mg GAE/100 g DM; TFC 1042.73 ± 49.95 vs 267.47 ± 6.38 mg QE/100 g DM; ORAC 39.48 ± 1.39 vs 11.09 ± 0.166 mmol TE/100 g DM.	Screening-level organ/tissue comparator evidence supporting separate interpretation of leaf and root fractions.	Microbiological characteristics were reported, but no stabilization route, validated storage/release specification, challenge study, or application-matrix performance was established.

Supplementary Table S1. Continued.

Panel A. Tissue-defined compositional and within-study comparator evidence (continued)

Reference and exact source locator	Crop and tissue-defined material	Postharvest state and replication	Stabilization or analytical condition	Quantitative endpoint and unit/basis	Evidence role in this review	Main claim boundary
[100] Dubrovska et al. (2026) DOI: 10.3390/foods15061009 Powder composition and water-related properties: Section 3.1 and associated tables; application context reported separately in Panel C.	White-cabbage processing by-products: leaf and core fractions maintained as distinct tissue-derived powders; unsteamed ("fresh-treatment") and steamed treatments analyzed separately.	Unsteamed ("fresh-treatment") and steamed leaf and core fractions were frozen, freeze-dried, milled, and analyzed separately. Independent industrial production-batch validation was not established.	Unsteamed and steamed tissues were freeze-dried, milled, and characterized for composition, water-related properties, functionality, color, and antioxidant-related endpoints.	Powder moisture 9.47-13.79%; water activity <0.2. All four powders contained >30 g TDF/100 g DW. Core powders showed higher reported mean K and P concentrations, whereas leaf powders showed higher reported fructose, glucose, and total-sugar concentrations. Pretreatment altered water absorption, solubility, color, and phenolic/antioxidant responses; values remained material- and treatment-specific.	Specification-supporting powder characterization showing tissue- and pretreatment-dependent behavior.	The reported ranges do not constitute a class-wide specification. Multi-batch variability, long-term storage, validated safety release criteria, and scale-up were NE.

Panel B. Stabilization and process-condition evidence

Panel-level boundary. The process studies support within-study condition selection; they do not establish safety release criteria, commercial throughput, storage-life validation, or application eligibility beyond the reported conditions.

Reference and exact source locator	Crop and tissue-defined material	Postharvest state and replication	Stabilization or analytical condition	Quantitative endpoint and unit/basis	Evidence role in this review	Main claim boundary
[104] Chantaro et al. (2008) DOI: 10.1016/j.lwt.2007.11.013 Results section (pp. 1989-1992).	Carrot peels from a ready-to-eat vegetable-processing stream; peel-rich root tissue.	Carrot peels were obtained as a by-product from a ready-to-eat vegetable-processing stream; the number of independent source lots and the elapsed pre-processing holding time were NR.	Blanching pretreatment and hot-air drying at 60-80 °C were evaluated before milling into dietary-fiber powder.	Initial peel moisture was approximately 1152 g water/100 g dry matter, as reported by the source. Blanching significantly affected fiber composition, WRC, and swelling, whereas drying temperature within 60-80 °C did not significantly affect hydration properties.	Specification-supporting evidence on the high initial water load and treatment-dependent hydration behavior of a defined carrot-peel fraction.	One processing-stream context. Final water activity, microbial release criteria, validated storage stability, independent batches, and food-matrix performance were NE or NR.

Supplementary Table S1. Continued.

Panel B. Stabilization and process-condition evidence (continued)

B2. Within-study comparison of white-cabbage outer-leaf drying arms

Common study conditions [132]. White-cabbage outer leaves from a Bangkok market were held at 4 °C, washed, steam-blanched for 1 min, cooled, sliced to 5 × 0.5 cm, and loaded at approximately 360 g per run. Drying was conducted at 80 °C in triplicate to 0.06-0.09 g/g DB; powders were milled at 18,000 rpm for 1 min, sieved to <450 µm, and vacuum-packed. Source locator: Figure 1 and Tables 1-3 (pp. 24-26); DOI 10.12982/cmujns.2017.0002.

Drying arm	Time (min)	WRC (g water/g)	Swelling (mL/g)	OHC (g oil/g)	Total glucosinolates (µmol/100 g DB; retention)	TPC (mg GAE/100 g DB; retention)	Within-study interpretation
Hot-air drying, 80 °C, 0.6 m/s	210	7.12 ± 1.23	11.63 ± 2.02	1.46 ± 0.19	1183.25 ± 58.01 (56.01 ± 4.84%)	1191.76 ± 33.39 (62.75 ± 3.25%)	Longest exposure; lower hydration and marker retention than vacuum-dried arms.
Vacuum drying, 80 °C, 5 kPa	135	11.53 ± 0.67	21.31 ± 1.35	1.32 ± 0.15	2003.36 ± 90.30 (94.60 ± 0.47%)	1559.58 ± 67.75 (82.08 ± 4.23%)	Shortest drying time; numerically highest mean WRC, swelling, glucosinolate retention, and TPC retention among the dried arms.
Vacuum drying, 80 °C, 10 kPa	175	10.34 ± 0.08	19.64 ± 1.28	1.21 ± 0.16	1943.08 ± 79.56 (91.77 ± 0.48%)	1544.32 ± 31.38 (81.34 ± 4.76%)	Higher absolute pressure lengthened drying relative to 5 kPa while retaining high hydration and marker values.
Steam injection, 1 min, then vacuum drying, 80 °C, 10 kPa	165	10.02 ± 1.16	17.45 ± 0.18	1.45 ± 0.07	1927.99 ± 69.12 (91.07 ± 0.64%)	1434.09 ± 40.69 (75.46 ± 2.40%)	Additional steam did not improve WRC over vacuum drying alone and yielded a numerically lower mean TPC retention than vacuum drying at 10 kPa without steam injection.
Low-pressure superheated-steam drying, 80 °C, 10 kPa	180	6.01 ± 0.19	9.75 ± 0.16	1.48 ± 0.16	1776.05 ± 67.50 (83.89 ± 1.41%)	1435.03 ± 34.27 (75.52 ± 2.63%)	Lowest hydration performance among tested arms; OHC remained comparable across arms.

Interpretation. Values are mean ± SD (n = 3). OHC did not differ significantly among drying schemes. Vacuum drying improved WRC and swelling relative to hot-air drying. The 5 kPa arm had the shortest drying time and the numerically highest mean glucosinolate and TPC retention among the dried powders; significance groupings should be interpreted from the source tables. These comparisons are valid within the shared material, loading, equipment, endpoint, and analytical basis.

Evidence role and claim boundary. Specification-supporting within-study process-comparison evidence; not route-level validation. The study used one laboratory-scale equipment/source context and triplicate laboratory-scale drying runs; independent source or production batches, validated microbial lethality, storage-life testing, and application-matrix evaluation were not reported.

Supplementary Table S1. Continued.

Panel C. Bounded application-matrix evidence

Reference and source locator	Material and application matrix	Formulation or test condition	Quantitative/application finding	Evidence role in this review	Main claim boundary
[101] Sudha et al. (2007)<br>DOI: 10.1016/j.foodchem.2006.12.016<br>Tables 2-5 (pp. 690-691).	Apple-pomace powder incorporated into a wheat-flour cake system.	Physical and sensory cake trials used 0, 10, 20, and 30% pomace, whereas composition and total-phenol analyses were additionally reported for a cake containing 25% pomace. Cake volume was measured with n = 4, texture with n = 8, composition with n = 3, and sensory evaluation used six panelists.	Cake volume decreased from 850 ± 11.18 cm^3 at 0% to 620 ± 9.15 cm^3 at 30% (n = 4); texture increased from 1.03 ± 0.18 to 1.46 ± 0.12 kg force (n = 8).<br>At 25% pomace: TDF 14.20 ± 1.04% DB, IDF 8.40 ± 0.79% DB, and SDF 5.80 ± 0.68% DB (n = 3).<br>Total phenols increased from 2.07 mg GAE/g in control cake to 3.15 mg GAE/g at 25%. Sensory overall-quality score was 4.0/5 at 20% and 3.0/5 at 30%.	Specification-supporting compositional evidence with bounded application-matrix performance testing; not full route-level validation.	One cake system and dose range. Enrichment was accompanied by a dose-related volume/texture penalty; validated safety, storage, scale-up, and cross-matrix performance were NE.
[100] Dubrovska et al. (2026)<br>DOI: 10.3390/foods15061009<br>Jelly formulation/optimization and application results.	A fixed powder mixture comprising 75% steamed cabbage leaves and 25% steamed cabbage cores was incorporated into pectin-based jelly formulations.	The concentration of the fixed steamed leaf–core powder mixture and the pectin concentration were varied in the study-specific formulation design; tissue ratio and pretreatment identity were held constant.	Powder concentration primarily affected jelly composition, including dietary fiber and phenolic-related endpoints, whereas pectin concentration primarily affected texture. Multi-response optimization identified 3.08% cabbage powder and 2.5% pectin as the study-specific optimum. Each jelly sample was prepared in duplicate as two separate batches, and sensory evaluation involved 37 potential consumers; the findings remain formulation- and matrix-specific. Application findings were interpreted together with the powder moisture, water-activity, TDF, functionality, and color data summarized in Panel A.	Specification-supporting powder evidence with bounded application-matrix testing; partial route-level performance evidence, not full route-level validation.	Single jelly matrix and study-specific optimization space. Two laboratory preparation batches were used per jelly sample, but independent industrial production-batch validation, long-term product stability, validated safety criteria, manufacturing scale-up, and cross-product reproducibility were not established.

Table-level claim boundary. The values are condition-specific evidence examples. They do not confer general food-grade status, validated safety, storage stability, commercial readiness, or route eligibility beyond the cited materials and experiments.

Study-specific source notes. [a]For Sudha et al. [130], the abstract reports 7.16 mg GAE/g for apple pomace, whereas Results Section 3.7 and Table 5 report 10.16 mg GAE/g; the value reported in Results Section 3.7 and Table 5 is reproduced here. [b]For Goyeneche et al. [131], the Methods section and Table 2 label mineral concentrations as g/100 g FW, whereas the abstract and Results text report mg/100 g; mg/100 g FW is reproduced here because this unit is internally consistent with the reported values and interpretation.

Abbreviations. DB, dry basis; DM, dry matter; FW, fresh weight; GAE, gallic acid equivalents; IDF, insoluble dietary fiber; NA, not applicable; NE, not evaluated; NR, not reported; OHC, oil-holding capacity; ORAC, oxygen radical absorbance capacity; QE, quercetin equivalents; SDF, soluble dietary fiber; TDF, total dietary fiber; TE, Trolox equivalents; TFC, total flavonoid content; TPC, total phenolic content; WRC, water-retention capacity.

Reference synchronization. Reference numbering follows the main manuscript. The complete entries used in this table are reproduced below for standalone readability.

References cited in Supplementary Table S1

[100] Dubrovska, A.; Galoburda, R.; Kruma, Z.; Ozola, L.; Straumite, E. Valorization of By-Products from White Cabbage (*Brassica oleracea* var. *capitata*) Processing. Foods 2026, 15, 1009. https://doi.org/10.3390/foods15061009.

[101] Sudha, M.L.; Baskaran, V.; Leelavathi, K. Apple pomace as a source of dietary fiber and polyphenols and its effect on the rheological characteristics and cake making. Food Chemistry 2007, 104, 686-692. https://doi.org/10.1016/j.foodchem.2006.12.016.

[102] Goyeneche, R.; Roura, S.I.; Ponce, A.G.; Vega-Gálvez, A.; Quispe-Fuentes, I.; Uribe, E.; Di Scala, K. Chemical characterization and antioxidant capacity of red radish (*Raphanus sativus* L.) leaves and roots. Journal of Functional Foods 2015, 16, 256-264. https://doi.org/10.1016/j.jff.2015.04.049.

[103] Tawatsinlapasorn, N.; Kuljarachanan, T.; Chiewchan, N.; Devahastin, S. Effects of Drying Techniques on Selected Functional Properties and Bioactive Compounds of Dietary Fiber from the Outer Leaves of Cabbage. Chiang Mai University Journal of Natural Sciences 2017, 16, 19-30. https://doi.org/10.12982/cmujns.2017.0002.

[104] Chantaro, P.; Devahastin, S.; Chiewchan, N. Production of antioxidant high dietary fiber powder from carrot peels. LWT - Food Science and Technology 2008, 41, 1987-1994. https://doi.org/10.1016/j.lwt.2007.11.013.
