# Supplementary material for: Fruit and Vegetable By-Products as Postharvest Tissue Fractions: A Raw-Material Framework for Plant-Based Food Development
Source: Plants (Basel). 2026 Aug 8;15(16):2423. doi: 10.3390/plants15162423 (PMC13516057; doi:10.3390/plants15162423)
Supplement: Supplementary file 1 [file plants-15-02423-s001.zip › plants-4407687_Supplementary_Table_S2.pdf]

Supplementary Table S2. Author-constructed paired examples showing how reporting completeness constrains evidence interpretation across the tissue-to-raw-material framework.

Purpose and provenance. All examples are hypothetical and author-constructed. They are not quotations, paraphrases, or reconstructed case summaries from identifiable studies. The reporting domains correspond to the minimum reporting checklist presented in Table 6 of the main manuscript. "Interpretation-ready reporting" means that material identity, process conditions, analytical basis, evidence limits, and claim boundary are transparent; it does not imply methodological validity, safety, regulatory eligibility, or application readiness. Numerical values and process conditions are illustrative and are not proposed specifications, legal thresholds, or universal acceptance criteria. Literature-derived quantitative evidence is provided separately in Supplementary Table S1.

Reading rule. The final column states the maximum interpretation supported by the reporting record. The actual evidence-use level also depends on study design, method performance, replication, and the observed results.

Panel A. Material identity and postharvest traceability

Reporting domain	Ambiguous or insufficient reporting example	Missing information and interpretive limitation	Interpretation-ready reporting example	Maximum supported interpretation and claim boundary
**Operational material status and intended route**	"Vegetable waste was used as an ingredient."	The generic term "waste" does not identify the operational material status, generation point, tissue source, segregation boundary, stabilization state, or intended route. It cannot support classification as a by-product, raw-material candidate, food-grade material, or legally eligible ingredient.	"Napa-cabbage outer leaves generated during trimming at a fresh-cut facility were segregated from mixed residues and recorded as a defined tissue fraction. They were operationally treated as a by-product and evaluated as a raw-material candidate for a powder route; no food-grade or legal-use status was inferred."	Supports operational classification and route scoping only. It does not establish safety, legal eligibility, specification compliance, or application readiness.
**Crop, taxon, organ/tissue identity, and segregation**	"Broccoli waste was dried and milled."	A crop-level label conflates leaves, florets, stalks, stems, and mixed trimmings. Missing taxon, organ/tissue identity, segregation status, and relevant biological covariates prevent tissue-specific interpretation of structure, composition, deterioration, and processability.	"Broccoli (*Brassica oleracea* var. *italica*) stalks remaining after floret separation were collected as a distinct stalk fraction. Leaves and florets were excluded, and cultivar and harvest maturity were recorded where relevant to material identity or composition. The fraction was processed independently."	Supports tissue-specific screening interpretation and prevents pooling across non-equivalent organs. The evidence level still depends on process definition, analytical methods, replication, and observed results.
**Generation point, lot identity, and postharvest quality history**	"Samples were collected from a market and dried."	The source facility, generation process, collection date, lot identity, elapsed time, holding temperature, mechanical damage, decay status, sanitation, transport, and time to stabilization are unknown. Handling effects cannot be separated from deterioration or source variability.	"Radish trimmings from lot RT-01 were collected at the fresh-cut line on the stated date within 15 min of cutting, held for 2 h at 4 °C in clean covered containers under documented handling conditions, inspected for mechanical damage and decay, washed using the stated procedure, and stabilized 2.5 h after generation."	Supports attribution of later measurements to the documented postharvest trajectory. It does not justify extrapolation to untested holding periods, seasons, lots, facilities, or transport conditions.

Supplementary Table S2. Continued.

Panel B. Stabilization, characterization, and specification

Reporting domain	Ambiguous or insufficient reporting example	Missing information and interpretive limitation	Interpretation-ready reporting example	Maximum supported interpretation and claim boundary
**Initial quality and route-relevant safety screening**	"The material was safe and suitable for food use."	This is an unbounded conclusion without a defined lot, intended route, hazard rationale, sampling plan, analytical method, unit, LOD/LOQ, acceptance criterion, jurisdiction, or condition of use. A fixed analyte panel may also be inappropriate across dissimilar tissues and routes.	"For the defined tissue fraction and intended route, relevant physicochemical, microbiological, and chemical hazards were selected using a documented risk-based rationale. The tested lot, sampling plan, analytical method, unit, LOD/LOQ, and acceptance criterion were reported for each parameter. Depending on the material and route, the panel may include moisture, water activity, microbial indicators, pesticide residues, heavy metals, mycotoxins, or soil-contact contaminants."	May support conditional safety-screening evidence for the tested lot and intended route only. It does not establish general food-grade status, jurisdiction-wide compliance, or safety for untested uses.
**Stabilization and preprocessing conditions**	"The material was dried and converted into powder."	The delay before stabilization, sorting or washing, pretreatment, equipment, loading, dimensions, temperature, time, pressure or airflow, endpoint, milling, and sieve size are absent. The process cannot be reproduced or compared reliably.	"The defined fraction was stabilized 2.5 h after generation, blanched at 90 °C for 2 min, cooled, loaded at 350 g per tray, hot-air dried at 60 °C and 1.0 m/s to measured endpoints of 8.0% moisture and water activity 0.42, and milled through a 500 µm sieve."	Supports reproduction of the stated stabilization process and condition-specific process comparison. It does not establish storage stability, microbial lethality, release suitability, or scale-up performance.
**Packaging, storage, and stability verification**	"The powder was stable during storage."	The package, atmosphere, temperature, humidity, duration, sampling schedule, monitored attributes, analytical methods, and predefined stability criteria are not reported. Low initial moisture or water activity alone does not establish shelf life.	"Powder was packed in oxygen-barrier pouches, stored at 25 °C and 60% relative humidity, and evaluated at 0, 4, 8, and 12 weeks for moisture, water activity, microbial indicators, color, odor, caking, and the predefined identity or quality marker. Stability acceptance criteria were specified before analysis."	Supports a stability statement only for the defined package, atmosphere, temperature, humidity, duration, sampling schedule, and measured endpoints. It does not establish stability under other distribution or use conditions.
**Analytical basis, batch variability, and provisional specification**	"The powder was antioxidant-rich and suitable as a functional ingredient."	The assay, extraction conditions, units, analytical basis, marker identity, method performance, batch variability, safety criteria, acceptance logic, and application-level function are undefined. Screening assays alone cannot establish a raw-material specification or ingredient claim.	"Screening assays were reported with extraction conditions, analytical method, unit, and basis. Identity- or process-relevant markers, moisture, water activity, particle-size distribution, soluble and insoluble fiber, microbiological indicators, and route-relevant contaminants were measured using validated or fit-for-purpose methods. Multiple independently produced batches were analyzed, batch variability was reported, and any provisional acceptance range was explicitly identified as preliminary. Application performance was evaluated separately."	May support a provisional raw-material-candidate specification when identity, method performance, batch variability, and acceptance logic are transparent. It does not establish application performance, commercial release specifications, or legal eligibility.

Supplementary Table S2. Continued.

Panel C. Application, route assignment, and regulatory fit

Reporting domain	Ambiguous or insufficient reporting example	Missing information and interpretive limitation	Interpretation-ready reporting example	Maximum supported interpretation and claim boundary
**Application-matrix performance**	"The by-product can be used in plant-based foods."	No target matrix, inclusion level, comparator, processing conditions, performance endpoints, sensory method, safety assessment, storage period, or acceptance threshold is defined. The claim generalizes beyond any tested application route.	"The stabilized powder was incorporated at 0%, 2%, 4%, and 6% (w/w) into a defined plant-based patty formulation using a matched control and fixed processing conditions. Hydration, cooking loss, texture, color, sensory acceptance, microbial quality, and refrigerated storage stability were evaluated using predefined methods and acceptance criteria."	Supports bounded application-matrix evidence for the tested formulation, processing conditions, inclusion levels, storage period, and endpoints. It does not support generalization to other products, processes, doses, or storage systems.
**Route assignment, downgrading, and exclusion**	"The best inclusion level was selected and unsuitable samples were rejected."	The intended route, decision criteria, comparator, acceptance limits, fallback option, lot-level disposition, and reasons for retention, downgrading, or exclusion are not transparent. The decision cannot be reproduced or audited.	"Predefined decision rules were applied after matrix testing. The 4% inclusion route was retained because all performance, sensory, and storage criteria were met. The 6% route was downgraded because bitterness and hardness exceeded the acceptance limits. Lots failing the predefined microbial criterion were excluded from route assignment."	Supports a route decision only for the tested matrix, inclusion range, processing conditions, storage context, and lots meeting the predefined criteria. Complete reporting alone does not establish route-level validation unless identity, safety, stability, process suitability, and application performance are all adequately demonstrated.
**Regulatory status and intended-use legal fit**	"The stabilized by-product was food-grade and legally usable."	Stabilization does not determine legal status. The statement omits jurisdiction, material identity, production process, intended technical effect, use level, target foods, anticipated exposure, history of food use, applicable pathway, labeling, and unresolved safety questions.	"For the proposed use in the stated jurisdiction, the material identity, production process, provisional specifications, intended technical effect, use level, target foods, anticipated exposure, history of food use, and relevant safety information were compiled. The potentially applicable regulatory pathway and remaining uncertainties were stated. No legal-eligibility or approval conclusion was made without a jurisdiction- and use-specific basis."	Supports identification of a possible regulatory pathway and unresolved evidence needs only. Legal eligibility remains jurisdiction-, material-, process-, and use-specific; examples may include GRAS, novel-food, food-additive, colorant, or food-contact pathways as applicable.

Notes. (1) All paired statements are author-constructed hypothetical examples and are not quotations, paraphrases, or evaluations of identifiable studies. (2) "Interpretation-ready reporting" means that the material, process, analytical basis, evidence limits, and claim boundary are transparent; it does not imply methodological validity, safety, legal eligibility, or application readiness. (3) Reporting depth should be adapted to the defined tissue fraction, intended route, study design, jurisdiction, evidence level, and claim strength. (4) The operational meanings of by-product, side-stream, tissue fraction, raw-material candidate, route assignment, downgrading, and exclusion from route assignment follow the main manuscript. Literature-derived quantitative evidence and study-specific claim boundaries are provided in Supplementary Table S1.
