# Supplementary material for: Fruit and Vegetable By-Products as Postharvest Tissue Fractions: A Raw-Material Framework for Plant-Based Food Development
Source: Plants (Basel). 2026 Aug 8;15(16):2423. doi: 10.3390/plants15162423 (PMC13516057; doi:10.3390/plants15162423)
Supplement: Supplementary file 1 [file plants-15-02423-s001.zip › plants-4407687_Supplementary_Table_S3.pdf]

Supplementary Table S3. Source-specific search strategies, search chronology, retrieval counts, record-management procedures, and manuscript citation-selection criteria used in the integrative evidence-mapping review.

Reporting scope. The complete database-specific strategies are reproduced as executed; line breaks were added only for readability. Source-level counts are raw records retrieved or captured before deduplication. Panel C reports the chronology of the final reproducibility audit, the consolidated identification-to-eligibility workflow, and the criteria used to construct the manuscript reference list. The numerical evidence flow is presented in Supplementary Figure S1.

Panel A. Bibliographic database searches

A1. Scopus (Elsevier)

Item	Details
**Information source / platform**	Scopus / Elsevier
**Search date**	28 July 2026
**Exact search strategy**	TITLE(by-product* OR byproduct* OR side-stream* OR pomace* OR trimming* OR "outer leaf" OR "outer leaves" OR "press cake" OR "press cakes" OR "fruit residue" OR "fruit residues" OR "vegetable residue" OR "vegetable residues" OR "processing residue" OR "processing residues" OR "fruit peel" OR "fruit peels" OR "vegetable peel" OR "vegetable peels" OR "peel by-product" OR "peel by-products" OR "peel residue" OR "peel residues"<br>)<br>AND<br>TITLE-ABS-KEY((fruit OR vegetable OR Brassicaceae OR cabbage OR broccoli OR cauliflower OR radish OR "napa cabbage") AND ((postharvest AND (quality OR handling OR storage OR deterioration)) OR ("post-harvest" AND (quality OR handling OR storage OR deterioration)) OR ((stabilization OR stabilisation) AND (quality OR safety OR storage OR drying)) OR (("raw material" OR "raw materials") AND (specification* OR safety OR stability OR ingredient*)) OR "physicochemical characterization" OR "compositional characterization" OR "techno-functional properties" OR "food ingredient" OR "food ingredients" OR "functional ingredient" OR "functional ingredients" OR (upcycl* AND (food OR ingredient* OR powder* OR fermentation OR coating* OR film*)))<br>)<br>AND<br>(DOCTYPE(ar) OR DOCTYPE(re)<br>)
**Coverage and limits**	Advanced document search; document types limited to Article and Review; no publication-year, language, or subject-area restriction.
**Records retrieved before deduplication**	n = 699
**Audit statement**	The complete query and displayed result count were verified against the supplied Scopus search record and export.

Supplementary Table S3. Continued.

A2. PubMed (NCBI)

Item	Details
**Information source / platform**	PubMed / NCBI
**Search date**	29 July 2026
**Exact search strategy**	(("by-product"[ti] OR "by-products"[ti] OR byproduct*[ti] OR "side-stream"[ti] OR "side-streams"[ti] OR pomace*[ti] OR trimming*[ti] OR "outer leaf"[ti] OR "outer leaves"[ti] OR "press cake"[ti] OR "press cakes"[ti] OR "fruit residue"[ti] OR "fruit residues"[ti] OR "vegetable residue"[ti] OR "vegetable residues"[ti] OR "processing residue"[ti] OR "processing residues"[ti] OR "fruit peel"[ti] OR "fruit peels"[ti] OR "vegetable peel"[ti] OR "vegetable peels"[ti] OR "peel by-product"[ti] OR "peel by-products"[ti] OR "peel residue"[ti] OR "peel residues"[ti]) AND (fruit[tiab] OR fruits[tiab] OR vegetable[tiab] OR vegetables[tiab] OR Brassicaceae[tiab] OR cabbage[tiab] OR broccoli[tiab] OR cauliflower[tiab] OR radish[tiab] OR "napa cabbage"[tiab]) AND (((postharvest[tiab] OR "post-harvest"[tiab]) AND (quality[tiab] OR handling[tiab] OR storage[tiab] OR deterioration[tiab])) OR ((stabilization[tiab] OR stabilisation[tiab]) AND (quality[tiab] OR safety[tiab] OR storage[tiab] OR drying[tiab])) OR (("raw material"[tiab] OR "raw materials"[tiab]) AND (specification*[tiab] OR safety[tiab] OR stability[tiab] OR ingredient*[tiab])) OR "physicochemical characterization"[tiab] OR "compositional characterization"[tiab] OR "techno-functional properties"[tiab] OR "food ingredient"[tiab] OR "food ingredients"[tiab] OR "functional ingredient"[tiab] OR "functional ingredients"[tiab] OR (upcycl*[tiab] AND (food[tiab] OR ingredient*[tiab] OR powder*[tiab] OR fermentation[tiab] OR coating*[tiab] OR film*[tiab])))) NOT preprint[pt]
**Coverage and limits**	No interface-applied date, language, or article-type filters. Preprints were excluded within the query using NOT preprint[pt].
**Records retrieved before deduplication**	n = 192
**Audit statement**	The exact submitted query was copied from the PubMed search-history record. The final Search Details/query translation was retained in the authors' audit files.

Supplementary Table S3. Continued.

A3. Web of Science Core Collection (Clarivate)

Item	Details
**Information source / platform**	Web of Science Core Collection / Clarivate Web of Science
**Search date**	Original search: 28 July 2026; verification rerun: 29 July 2026
**Exact search strategy**	TI=(by-product* OR byproduct* OR side-stream* OR pomace* OR trimming* OR "outer leaf" OR "outer leaves" OR "press cake" OR "press cakes" OR "fruit residue" OR "fruit residues" OR "vegetable residue" OR "vegetable residues" OR "processing residue" OR "processing residues" OR "fruit peel" OR "fruit peels" OR "vegetable peel" OR "vegetable peels" OR "peel by-product" OR "peel by-products" OR "peel residue" OR "peel residues") AND TS=((fruit OR fruits OR vegetable OR vegetables OR Brassicaceae OR cabbage OR broccoli OR cauliflower OR radish OR "napa cabbage") AND (((postharvest OR "post-harvest") AND (quality OR handling OR storage OR deterioration)) OR ((stabilization OR stabilisation) AND (quality OR safety OR storage OR drying)) OR (("raw material" OR "raw materials") AND (specification* OR safety OR stability OR ingredient*)) OR "physicochemical characterization" OR "compositional characterization" OR "techno-functional properties" OR "food ingredient" OR "food ingredients" OR "functional ingredient" OR "functional ingredients" OR (upcycl* AND (food OR ingredient* OR powder* OR fermentation OR coating* OR film*))))
**Coverage and limits**	Advanced Search - Query Builder; all available editions; no document-type, publication-year, language, or subject-area restriction.
**Records retrieved before deduplication**	n = 563
**Audit statement**	The raw export was generated on 28 July 2026. The exact query was rerun on 29 July 2026 solely to verify the syntax and displayed result count; the original exported record set was retained.

Supplementary Table S3. Continued.

Panel B. Google Scholar supplementary search

Handling and counting rule. All searches were run in Microsoft Edge InPrivate mode while logged out, using relevance ordering and no date or language restriction. Patents and citation-only results were excluded. The prespecified depth was the first 50 accessible relevance-ranked results per query; all available results were recorded when fewer than 50 were accessible. Only records inspected and captured under this source-level retrieval rule, not Google Scholar estimated hit counts, were included in the pre-deduplication record pool. These records then entered the common deduplication and title/abstract eligibility-screening workflow.

Query and purpose	Exact query displayed	Search date	Records inspected and captured / last rank
**GS_Q1.** Fruit by-products—broad title-based supplementary search	allintitle: fruit by-product OR by-products OR byproduct OR byproducts	2026-07-28	**50 / 50**
**GS_Q2.** Pomace stabilization, drying, powder, food-ingredient, upcycling, and valorization pathway	allintitle: pomace ingredient OR powder OR upcycling OR drying OR stabilization OR stabilisation OR valorization	2026-07-28	**50 / 50**
**GS_Q3.** Vegetable by-products—broad title-based supplementary search	allintitle: vegetable by-product OR by-products OR byproduct OR byproducts	2026-07-28	**50 / 50**
**GS_Q4.** Brassicaceae crop-specific by-products—targeted title-based supplementary search	allintitle: by-product Brassicaceae OR cabbage OR broccoli OR cauliflower OR radish OR "napa cabbage"	2026-07-28	**43 / 43**
**GS_Q5.** Outer leaves and leaf-fraction evidence—targeted title-based supplementary search	allintitle: cabbage OR broccoli OR cauliflower OR "napa cabbage" OR drying OR powder OR ingredient OR fiber OR fibre OR stabilization OR stabilisation OR valorization "outer leaves"	2026-07-29	**34 / 34**

Supplementary Table S3. Continued.

Panel C. Process chronology, record management, evidence-flow accounting, and citation-set construction

Stage	Procedure	Verified result
**Process chronology**	The evidence synthesis was developed iteratively during manuscript preparation. The source-specific searches reported in this table were executed or verified on 28–29 July 2026 to establish the final reproducible source pool and exact evidence-flow counts. After the final source search, the source records were consolidated and reconciled with the retained title/abstract and full-text assessment logs. Duplicate-resolution records, eligibility coding, and author verification of the consolidated evidence-flow workbook were finalized on 30 July 2026.	Final source search completed on 29 July 2026; consolidated evidence-flow record finalized and author-verified on 30 July 2026.
**Record import and deduplication**	Raw records from Scopus, PubMed, Web of Science Core Collection, and Google Scholar were retained with source provenance. Duplicate candidates were resolved sequentially using exact DOI, exact PMID, Scopus EID or Web of Science UT, and normalized title + year + first author, followed by author checking. Raw rows were not deleted; duplicate relations and representative records were logged.	1,681 records identified; 679 duplicate records removed; 1,002 unique records remained.
**Title/abstract screening**	Unique records were screened using the title, available abstract or Google Scholar snippet, and bibliographic information. Records judged potentially eligible or uncertain proceeded to full-text assessment.	1,002 records screened; 33 excluded; 969 reports sought for retrieval.
**Full-text retrieval and eligibility**	Full texts were retrieved and assessed against the predefined material, tissue-identity, tissue-to-raw-material, and synthesis-domain criteria. Each excluded report was assigned one mutually exclusive primary reason (FT1-FT6).	969 reports retrieved and assessed; 0 not retrieved; 97 excluded at full text; 872 eligible reports retained in the evidence bank. FT1-FT5 = 0; FT6 = 97.
**Citation searching**	Backward and forward citation searching were not performed; therefore, no citation-search records were added to the master pool.	Backward = 0; forward = 0.
**Human verification**	H.J.W. completed and confirmed deduplication, title/abstract screening, full-text assessment, exclusion-reason assignment, and reference checking. A.-J.C. verified the recorded screening and full-text eligibility outcomes; discrepancies were resolved by discussion.	Author verification completed on 30 July 2026.
**Citation-set construction**	Evidence-bank eligibility and individual manuscript citation were separate decisions; no second eligibility screen reduced 872 reports to 134 references. Evidence-bank reports were cited when they directly and non-redundantly supported a specific claim, table, figure, or synthesis domain. Where several eligible reports supported the same point, priority was given to sources with clearer crop and tissue identity, postharvest or processing conditions, analytical methods and units, explicit claim boundaries, and direct relevance to the statement being supported. Primary studies were generally prioritized for quantitative and process-specific claims, broader reviews for cross-cutting synthesis, and official sources for methodological, regulatory, or policy statements. The final reference list also contained contextual and reporting-policy sources used where needed. Eligible reports not cited individually remained in the evidence bank and were not treated as additional exclusions.	872 eligible reports were retained in the evidence bank, and 134 manuscript references were cited and audited. Individual citation was not treated as an additional eligibility-screening stage.

Notes. (1) Records are bibliographic entries evaluated at the title/abstract stage; reports are full-text documents assessed for eligibility. (2) Source-level retrieval counts are before deduplication. Google Scholar counts are the records inspected and captured under the prespecified source-level retrieval-depth rule; estimated hit counts were retained only as internal audit metadata and were not used in Supplementary Figure S1. (3) The Web of Science query was originally exported on 28 July 2026 and rerun on 29 July 2026 solely to verify the exact syntax and displayed count. (4) Material-scope, tissue-identity, and clearly non-material-focused records were resolved primarily during title/abstract screening; consequently, all 97 reports excluded after full-text assessment were assigned FT6. (5) The evidence-bank count (n = 872) and manuscript reference-list count (n = 134) are different accounting units. The reference list was not generated by a second eligibility screen; its construction is described in Panel C.
